# Improvements in identification and quantitation of alkylated PAHs and forensic ratio sourcing

**DOI:** 10.1007/s00216-020-03127-0

**Published:** 2021-01-28

**Authors:** Christine C. Ghetu, Richard P. Scott, Glenn Wilson, Rachel Liu-May, Kim A. Anderson

**Affiliations:** 1 Department of Environmental and Molecular Toxicology, Oregon State University, Corvallis, OR 97331 USA; 2 Department of Chemistry, Oregon State University, Corvallis, OR 97331 USA

**Keywords:** Forensic ratios, Alkylated PAHs, Forensic sourcing, Polycyclic aromatic hydrocarbons, Triple quadrupole mass spectrometry, Gas chromatography

## Abstract

Parent and alkylated polycyclic aromatic hydrocarbons (PAHs) are present in a number of different sources in varying proportions depending on the source material and weathering. This range of PAH sources can make it difficult to determine the origin of exposure(s). Ratios of alkylated and parent PAHs have been applied as a forensic tool to distinguish between different sources. However, few studies have examined PAH ratios comprehensively as indicators for sourcing beyond a single study area or matrix type. In this paper, we introduce an expanded analytical method based on ASTM D7363-13a which we adapted for a gas chromatography triple quadrupole mass spectrometry instrument. The modifications increase selectivity and sensitivity compared to the ASTM method. We added five alkylated series to the method. This method has then been applied to 22 independent forensic ratios. We evaluated the method and the forensic ratios with certified reference materials and known environmental samples. This analytical method and thirteen PAH ratios were found to accurately predict sources of PAHs.

**Supplementary Information:**

The online version contains supplementary material available at 10.1007/s00216-020-03127-0.

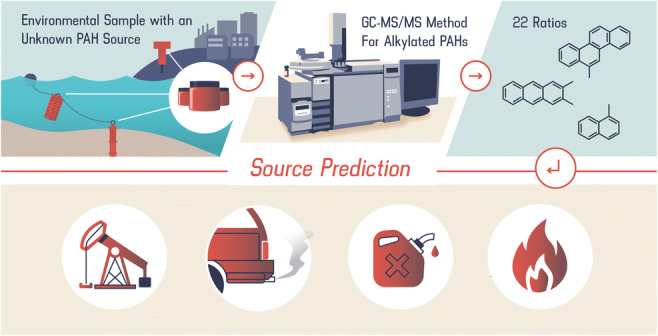

## Introduction

**Fig. 1:**
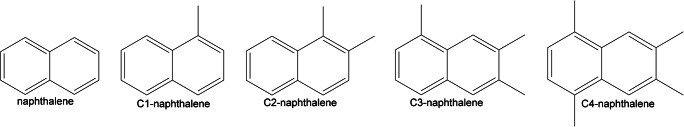
Example of some naphthalene alkylated series

Polycyclic aromatic hydrocarbons (PAHs) are a class of widespread organic contaminants arising from both natural and anthropogenic sources such as oil seeps, wildfires, and fossil fuel combustion. PAHs are defined as having at least two fused aromatic rings, and are mainly semi-volatile. These compounds can exist as unsubstituted, parent compounds, or with the addition of substituted functional groups, subclasses like alkylated, oxygenated, nitrated, and hydroxy PAHs. One subclass of PAHs, known as alkylated PAHs, are defined as having hydrocarbon functional groups substituted onto the parent structure. These substituents can range from one hydrocarbon group, known as C1-PAHs, to much larger hydrocarbon groups (see Fig. 1). Depending on the source, alkylated PAHs can represent more than 50% of the total PAH mass [1–3].

All PAHs can come from petrogenic or pyrogenic sources. Petrogenic sources originate from hydrocarbons within rock, and are generated by geological conditions including time and temperature over millions of years. Pyrogenic sources are derived through high temperature, oxygen derived combustion, or pyrolysis of organic matter. Classifying PAHs as either petrogenic or pyrogenic can therefore provide some information in identifying different sources contributing to PAHs at contaminated sites. Parent and alkylated PAHs are found in varying proportions based on the source material and weathering.

Many ratios of PAH compounds, including both parent and alkylated, have been applied as a forensic tool to distinguish PAH sources [4–6]. Different PAH proportions develop due to PAH physical chemical properties within the source. Environmental conditions, such as temperature, contribute to the alteration of PAH proportions with weathering. These PAH ratios have been observationally developed based on known sources. However, in most studies, only a few PAH ratios are used to distinguish between PAH sources. Additionally, in many case studies, the source was mixed, so evaluation of a specific PAH ratio’s effectiveness in source prediction was not definitive. The lack of consistent evaluation has led to uncertainty in the strength of specific PAH ratios to successfully distinguish different sources. Using a converging lines of evidence approach, with many PAH ratios that encompass a range of molecular weights, a more robust interpretation of environmental sources could be developed [6]. To date, a few studies have attempted a comprehensive examination of PAH ratios as indicators for source. This paper builds on the approach first introduced by Yunker et al. [6], Stogiannidis and Laane [5], and Tobiszewski and Namieśnik [4], in which many different PAH ratios are reviewed for their ability to accurately distinguish between petrogenic and pyrogenic sources, natural and anthropogenic sources, and PAHs in air, water, and sediment porewater.

In this study, we introduce an expanded and modified analytical method based on ASTM D7363-13a [7] using a gas chromatography triple quadrupole mass spectrometry instrument (GC-MS/MS). The ASTM method uses a single quadrupole gas chromatography instrument, and was developed for sediment porewater to meet the goals of the US EPA narcosis model for protecting benthic organisms in PAH-contaminated sediments [7]. ASTM D7363-13a also uses NIST SRM 1991 – Mix Coal Tar/Petroleum Extract in Methylene Chloride as a calibration solution for the method. Using a real-world, oil-based standard introduces matrix effects and contains chemicals that have not yet been identified. However, most alkylated PAH standards are not available, and trying to make a solution containing all possible alkylated PAHs would be expensive and time consuming. Instead, alternative approaches need to be used, making method development for alkylated PAHs difficult. In order to address limitations in the original ASTM method, additional alkylated PAHs applicable for air, water, and sediment porewater matrices are needed, in addition to better sensitivity and accuracy of the analytical technique.

The triple quadrupole is more selective than a single quadrupole mass spectrometer, and increased selectivity generates a lower background leading to decreased signal bias. Increased selectivity also means a lower voltage is required to detect higher molecular weight PAHs. Lower voltages are highly advantageous in mass spectroscopy methods because it decreases non-linear calibration for lower molecular weight compounds and allows for a larger calibration range. A larger calibration range is often beneficial for environmental samples since target concentrations can often span several orders of magnitude. Additionally, a triple quadrupole method removes interferences that often lead to false signals. Reduction of false signals is especially important when analyzing alkylated PAHs, where multiple peaks are integrated together to capture the entire alkylation profile for an individual C-PAH series.

The objectives of this study were to (1) expand, modify, and validate a method for alkylated PAHs by GC-MS/MS; (2) evaluate forensic PAH ratios and develop a weighted score for source identification; and (3) combine these two approaches and apply them to certified reference materials and known environmental samples.

## Materials and methods

### Standards and reagents

Analytical grade standard reference materials (SRM) were obtained from National Institute of Standards and Technology (NIST) (Gaithersburg, MD) including SRM 1991 – Mix Coal Tar/Petroleum Extract in Methylene Chloride, SRM 2779 – Gulf of Mexico Crude Oil, SRM 1580 – Organics in Shale Oil, SRM 1582 – Petroleum Crude Oil, SRM 1597a – Complex Mixture of Polycyclic Aromatic Hydrocarbons from Coal Tar, and SRM 1975 – Diesel Particulate Extract. All solvents used were Optima grade (Fisher Scientific, Pittsburg, PA) or equivalent. All deuterated surrogates were obtained from CDN Isotopes (Pointe-Claire, Quebec, Canada). For environmental samples undergoing extraction, these deuterated standards were added to assess extraction recovery and efficiency. Five deuterated surrogates were selected for this method: naphthalene-d_*8*_, acenaphthylene-d_*8*_, phenanthrene-d_*10*_, fluoranthene-d_*10*_, and chrysene-d_*12*_, prepared in ethyl acetate and added at 500 pg/μl as an internal surrogate for quantification of compounds of similar structure and chemical behavior. Perylene-d_*12*_ was selected for use as an instrumental internal standard, prepared in isooctane and added at 500 pg/μl due to its reproducible signal and similar structure to larger PAHs. This approach is similar to that applied in Anderson et al. for development of quantitative separation of 62 PAHs [8]. PAH compound and forensic ratio abbreviations are listed in Table 1.

**Table 1:** PAH and forensic ratio abbreviations

**A**	Anthracene	**ghi**	Benzo[ghi]perylene
**P**	Phenanthrene	**BeP**	Benzo[e]pyrene
**FL**	Fluoranthene	**BbF**	Benzo[b]fluoranthene
**PY**	Pyrene	**BkF**	Benzo[k]fluoranthene
**BaA**	Benz[a]anthracene	**DA**	Dibenz[ah]anthracene
**Nap**	Naphthalene	**Ay**	Acenaphthylene
**F**	Fluorene	**Ace**	Acenaphthene
**Ch**	Chrysene	**Per**	Perylene
**Trp**	Triphenylene	**IP**	Indeno[1,2,3-c,d]pyrene
**D**	Dibenzothiophene	**L/H**	Low PAHs/High PAHs
**BaP**	Benzo[a]pyrene	**LPAH/HPAH**^**a**^	Low molecular weight PAHs/high molecular weight PAHs

^a^Low molecular weight PAHs versus high molecular weight PAHs refers to compounds listed in the LPAH/HPAH ratio in Table 3. LPAH have a molecular weight of 128–178 g/mol; HPAH have a molecular weight of 202–276 g/mol

### Sample preparation

#### Standards

To develop our expanded method, we used calibration standards prepared through serial dilution of NIST SRM 1991 ranging from 1:1 to 1:16,000. This provided a concentration range of 17–28,000 pg/μl based on C2-naphthalenes. To validate our method and list of forensic PAHs ratios, we selected five NIST SRM solutions. SRMs 2779, 1580, and 1582 were prepared gravimetrically in dichloromethane and then serially diluted. SRM 1597a was diluted 50-fold in toluene. SRM 1975 was diluted 10-fold in dichloromethane.

Depending on the SRM, there were different numbers of known mass fractions of alkylated PAHs that could be used to later verify the modified, expanded analytical method (see Table 2). Using the certified and reference mass fraction values provided by NIST for SRMs 2779, 1582, 1580, 1597a, and 1975, expected concentrations for all parent and alkylation series were determined. 95% confidence levels were calculated for reference mass fraction values to take into consideration the expanded uncertainty. Expected concentrations were then compared to concentrations obtained from the analytical method to obtain the percent recovered.

**Table 2:** Summary of samples tested for alkylated PAH method and forensic ratios

Sample	Sample matrix	Source	Classification	NIST PAH mass fractions
SRM 2779	Oil	Crude oil	Petrogenic	N, C1-C4N, F, C1-C3F, P, C1-C4P&A, D, C1-C3D, FL, C1-C2FL&PY, Ch, Trp, C1-BaA&Ch&Trp, C2- BaA&Ch&Trp
SRM 1582	Oil	Crude oil	Petrogenic	N, C1-C4N, F, C1-C3F, P, C1-C4P&A, D, C1-C3D, FL, C1-C2FL&PY, Ch, Trp, C1-BaA&Ch&Trp, C2- BaA&Ch&Trp
SRM 1580	Oil	Shale oil	Mixed	N, P, FL, Ch, Trp
SRM 1597a	Slurry	Coal tar	Pyrogenic	N, C1-N, C2-N, F, P, C1-P&A, C2-P&A, D, C1-D, FL, C1-FL&PY, Chr, Trp, C1- BaA&Ch&Trp
SRM 1975	Slurry	Diesel particulate	Pyrogenic	N, C1-N, F, P, C1-P&A, C2-P&A, FL, C1-FL&PY, Ch, Trp
DWH	Water	Crude oil	Petrogenic	N/A
St. Helens, OR	Air	Creosote	Pyrogenic	N/A
St. Helens, OR	Water	Creosote	Pyrogenic	N/A
St. Helens, OR	Sediment porewater	Creosote	Pyrogenic	N/A
Chamber	Air	Fire	Pyrogenic	N/A

#### Known PAH source samples

Three sets of environmental samples were selected for further method and forensic ratio validation: six water samples for petrogenic signatures from the explosion of the Deepwater Horizon (DWH) oil well drilling platform in 2010; air, water, and sediment porewater samples for pyrogenic signatures from the location of the former Pope and Talbot Inc. and St. Helens Creosoting Company operations in St. Helens, OR; and finally, two samples collected from a fire smoke chamber for pyrogenic signatures.

Samples from DWH and St. Helens, OR, were obtained using low-density polyethylene (LDPE) passive sampling techniques. Sampling methodology and lab processing for DWH samples followed procedures as described by Allan et al. [9]. Sampling methodology and lab processing in St. Helens, OR, followed procedures as described by Minick and Anderson [10]. The two sediment porewater depths are designated shallow porewater (7.6–20 cm below the mudline) and deep porewater (61–74 cm below the mudline). Samples from the fire smoke chamber were obtained using silicone wristbands. Sampling methodology and lab processing for the chamber study followed procedures as described by Anderson et al. [11].

### QC samples

We analyzed instrument blanks (*n*-hexane) and calibration verifications at the beginning and end of each set of samples. All continuing calibration verifications were verified at ± 30% of the true concentration for ≥ 70% of analytes.

Any potential passive sampling background signal due to matrix effects or instrument noise was evaluated by the analysis of LDPE extraction blanks, laboratory processing blanks, and instrument blanks. The LDPE extraction blank consisted of one cleaned and extracted LDPE passive sampling strip. The laboratory processing blank consisted of one cleaned LDPE passive sampling strip that underwent all of the same laboratory processing as environmental samples. This quality control sample is used for sample concentration correction of environmental samples via background subtraction. The instrument blank was *n*-hexane solvent.

To create a matrix positive control sample, 10 μl of SRM 1991 was spiked into a strip of LDPE to yield a 1:100 dilution in final concentration. The LDPE strip was sealed, processed, and extracted as outlined in Minick and Anderson [10].

We used a 500 pg/μl calibration standard solution from another PAH method in our laboratory [8] to cross check our modified and expanded method. The calibration standard solution contains 43 individual parent and 19 individual alkylated PAHs, and will be referred to in the rest of the paper as “in-house standard.” The in-house standard was analyzed using the modified, expanded alkyl PAH method using both full scan and multiple reaction monitoring (MRM). Using this cross check approach, we identified compound interferences that would affect percent recoveries for each alkylation series. Instrument concentrations were determined by using the total response for each alkylation series. Expected concentrations were determined by adding up all analytes from the in-house standard corresponding to a given alkylation series. Expected concentrations were then compared to concentrations obtained from the alkylated PAH analytical method to obtain the percent recovered.

### GC-MS/MS

Instrumental analysis was performed on an Agilent 7890B gas chromatograph with an Agilent 7000C triple quadrupole mass spectrometer and J&W Scientific select PAH column (CP7462), 30 m × 0.25 mm × 0.15 μm. Instrument modifications were made to improve analytical performance in alkylated PAH analysis [7]. One microliter of sample was injected onto a 4-mm Agilent splitless single-taper liner packed with a small amount of deactivated glass wool in pulsed splitless mode using an injection temperature of 320 °C, purge flow to split valve 25 ml min^−1^ at 0.7 min, thermal auxiliary two (MSD transfer line) heater at 320 °C, and source temperature at 340 °C. An oven program of a hold at 60 °C for 1 min, ramping 40 °C min^−1^ to 180 °C, 3 °C min^−1^ to 230 °C, 1.5 °C min^−1^ to 235 °C, 15 °C min^−1^ to 280 °C min^−1^, hold for 10 min, ramp 6 °C min^−1^ to 298 °C, and a final ramp of 16 °C min^−1^ to 350 °C with a 4-min hold at 350 °C was used. Helium carrier gas was held at a constant flow rate of 2.0 ml/min. The triple quadrupole collision cell helium quench gas was set to 2.25 ml min^−1^ with nitrogen collision gas at 1.5 ml min^−1^. The total run time for the analysis was 47.25 min. Complete GC-MS/MS instrument conditions are appended in Supplementary Information (ESM) Table S2.

Parent PAH concentrations used for ratio calculations were obtained using our existing analytical method as described in Anderson et al. [8] and referred to in this paper as “in-house 62 PAH method”. The method uses modified GC-MS/MS, on an identical system to our expanded, modified alkylated PAH method to achieve quantitative separation of 62 PAHs (43 individual parent and 19 individual alkylated PAHs).

#### Optimized detection

Alkylation series separation was optimized through an iterative cycle of GC oven temperature profile adjustments based on the method developed by ASTM D7363-13a [7] and the in-house 62 PAH method [8]. Gas flow rates and temperature parameters were evaluated by making changes in flow, temperature, or ramp rate, and examining the effect on chromatographic separation and alkylation pattern shape. Final parameters include specifically defined ramp rates and ranges as well as inclusion of several isothermal holds to improve PAH separation. Optimization of the collision energy used for each alkylation series was determined through an iterative cycle of collision energy adjustments to achieve the highest response of the secondary ion [8]. Collision energies are appended in ESM Table S2.

### Data analysis

GC-MS/MS data was analyzed using MassHunter Quantitative Analysis v.B.06.00 SP1 build 6.0.388.1 (Agilent Corp. Wilmington, DE) software. In GC-MS/MS MRM, each analyte was positively identified by relative retention time and the ratio of the primary ion to secondary ion transition. Our criterion for transition ion ratios is that they must occur within ± 30% of expected relative response ratio in order to be considered confirmatory. Analytes, surrogate compounds, and the internal standard are identified and quantified as pg/μl concentration and corrected for surrogate compound recovery by the MassHunter software. Surrogate compound concentrations are calculated based on a ratio of relative response factors to the internal standard, which can be affected by different matrices and extraction methods. Analyte concentrations are based on a ratio of relative response factors to their specified surrogate compound (appended in ESM Table S3).

### Method calibration and validation

Alkylated PAH series were quantified using internal standard calibration, using on average 6 point calibration curves (ESM Table S3). Extracted ion chromatograms (EICs) were used to calibrate the method for each alkylation series. GC-MS/MS method detection limits were assessed through pattern recognition (see ESM Figs. S1-S6) of a peak series with a minimum signal to noise ratio greater than 3:1. Limits of quantitation (LOQs) for alkylation series were calculated as three times the background concentrations from analysis of a matrix blank. If alkylation series in the matrix blank were not detected, LOQs were determined from seven to nine replicates of two test concentrations of SRM 1991 (1:20 and 1:200). LOQs were then calculated by multiplying the standard deviation calculated from the replicate analysis by the Student *T* score for the 99% confidence interval (e.g., *n* = 7, Student *T* score: 3.143 or *n* = 9, Student *T* score: 2.896). Detection limits were based on an entire alkylation series, rather than the ASTM D7363-13a [7] approach of using a single isomer. Inter-day instrument analysis of precision and accuracy was performed by diluting NIST SRM 1991 and analyzing the solution seven times. Intra-day instrument analysis of accuracy and precision was performed by diluting NIST SRM 1991 and analyzing the solution in triplicate over 3 days (see ESM Table S4).

### Forensic ratios

PAH forensic ratios were compiled through a series of comprehensive literature reviews [4–6]. A total of 22 independent PAH ratios including parent PAHs, alkyl PAHs, and heterocyclic sulfur compounds were identified. Using available literature information for each ratio, a range of values for petrogenic and pyrogenic sources were listed (see Table 3). We then evaluated PAH ratios based on frequency in the literature, how often the ratio was successful in source prediction, how many environmental matrixes had been tested with the ratio, and how stable the ratio was under various environmental conditions. Based on these results, ratios were binned into three categories: strong, medium, and weak. The bins were given a simple weighted score of 1, 2, or 3, with 3 being successful and well tested, and 1 being less tested and less successful as identified by the authors of that study. The 22 forensic ratios used and their associated weighted scores are listed in Table 3.

**Table 3:** 22 forensic ratios for petrogenic and pyrogenic source and associated weighted scores

Abbreviation^a^	Ratio	Pyro ratio	Petro ratio	Pyro score	Petro score	Ref^b^	PAH ratio category
**A0/PA0**	A / A + P	> 0.1	< 0.1	3	3	2, 3	Low molecular weight PAHs
**P0/A0**	P / A	< 5	> 30	3	3	1	Low molecular weight PAHs
**PA1/PA0**	C1 P&A / P + A	< 1	> 1.5	1	2	1	Low molecular weight PAHs
PA0/PA01	P + A / P + A + C1 P&A	> 0.5	≤ 0.4	1	1	2	Low molecular weight PAHs
FL0/PY0	FL / PY	> 1	≤ 0.5	1	1	1	4-ringed PAHs
FL0/FLPY	FL / FL + PY	> 0.5	< 0.4	1	2	2, 3	4-ringed PAHs
**FLPY0/FLPY01**	FL + PY / FL + PY + C1 FL&PY	> 0.5	< 0.5	3	3	2	4-ringed PAHs
FLP1/PY0	C1 FL&PY / PY	~ 0.3	~ 4	1	1	1	4-ringed PAHs
FLP1/FLPY0	C1 FL&PY / FL + PY	< 1	> 1	2	1	1	4-ringed PAHs
FLPY/(P2 + P3 + P4)	FL + PY / (C2 + C3 + C4) P&A	< 0.3	> 9	1	1	1	4-ringed PAHs
BaA/Ch0	BaA / Ch	≥ 0.5	< 0.25	1	1	1	4-ringed PAHs
BaA/228	BaA / BaA + Ch + Trp	> 0.35	< 0.2	1	1	2, 3	4-ringed PAHs
D2/P2	C2 D / C2 P&A	< 0.4		1	N/A	1	Sulfur compounds
D3/P3	C2 D / C3 P&A	< 0.4		1	N/A	1	Sulfur compounds
PY0/BaP	PY / BaP	< 10	≥ 10	1	1	1	4–5 ringed PAHs
IP/ghi	IP / ghi	> 1	< 0.25	1	2	1	5–6 ringed PAHs
IP/IP + ghi	IP / IP + ghi	> 0.5	< 0.2	1	1	2, 3	5–6 ringed PAHs
BeP/BaP	BeP / BaP	< 1		1	N/A	1	5–6 ringed PAHs
∑alkyl/PAHs	(C1 + C2 + C3 + C4) Nap + (C1 +C2 +C3) P&A + (C1 +C2) FL&PY + C1 BaA&Ch&Trp / Nap + P + A + FL + PY + BaA + Ch	< 1	> 2.3	2	1	1	Sum alkyl/parent PAHs
L/H	P + A + FL + PY / BaA + Ch + BkF + BaP + IP + DA + ghi	< 1	> 1	1	1	1, 3	Low MW/high MW PAHs
LPAH/HPAH	Nap + Ace + Ay + F + P + A / FL + PY + BaA + Ch + BbF + BkF + BaP + IP + DA + ghi	< 0.4	> 2.3	1	1	1	Low MW/high MW PAHs
**Pyrogenic index**	Ay + Ace + A + FL + PY + BaA + BbF+ BkF + BeP + BaP + Per + ghi + IP + DA / (C1 + C2 + C3 + C4) Nap + (C1 + C2 + C3 + C4) P&A + (C1 +C2 +C3 + C4) D + (C1 + C2 + C3 + C4) F + (C1 + C2 + C3 + C4) BaA & Ch & Trp	0.8 ⟷ 2	< 0.05	2	2	1	Unsubstituted 3–6 ringed/five-target alkylated PAHs

^a^Abbreviations in bold indicate the ratio is definitive of a source

^b^1. Stogiannidis, E.; Laane, R.; Source characterization of polycyclic aromatic hydrocarbons by using their molecular indices: an overview of possibilities. In *Review of Environmental Contamination and Toxicology*; Whitacre, D.M., Ed.; Springer: New York, NY, 2014; 234, 49–177

2. Yunker, M.B.; Macdonald, R.W.; Vingarzan, R.; Mitchell, R.H.; Goyette, D.; Sylvestre, S.; PAHs in the Fraser River Basin: a critical appraisal of PAH ratios as indicators of PAH source and composition. *Org Geochem* 2002; 33, 489–515

3. Tobiszewski, M.; Namiesnik, J. PAH diagnostic ratios for the identification of pollution emission sources. *Environ Pollution* 2012; 162, 110–119

Utilizing the concentrations obtained from our expanded, modified alkylated PAH method and in-house 62 PAH method, ratios were calculated for all samples. Resulting ratio values were then sorted into either a petrogenic or pyrogenic category, and the corresponding weighted score was applied. If a ratio value fell between petrogenic and pyrogenic ranges, the ratio was considered mixed and a weighted score was not applied.

After calculating each ratio value and applying the weighted score, the sum score for pyrogenic and petrogenic categories was calculated. If the differences between the two summed scores were equal to or less than 2, the sample was predicted to be mixed in source. If the petrogenic and pyrogenic sum score differences were greater than 2, the sample was assigned one source as appropriate. With the exception of LPAH/HPAH and pyrogenic index ratios, PAH ratios were not calculated if one of the parent compounds or alkylation series was below detection limits.

## Results

### Method validation

For all alkylation series, a linear regression calibration with 1/x weighing was applied, with resulting coefficients of determination (*r*^2^) > 0.99. LOQ concentrations for each analyte and alkylation series are listed in Table 4. To the authors’ knowledge, these are the lowest reported instrument LOQs for alkylated PAH series. For method inter-day instrument analysis, the average percent recovery for all alkylation series was 98.9% (82.6–120%) with an average relative standard deviation of 4.67% (0.80–19.1%). Intra-day instrument analysis across 3 days for all alkylation series yielded an average percent recovery of 98.1% (74.4–118%) with an average relative standard deviation of 4.48% (0.60–15.6%). Average concentrations, percent recoveries, and relative standard deviation percentages for all alkylation series can be found in ESM Table S4.

**Table 4:** Expanded alkylated PAH method limits of quantitation

Analyte	LOQ (pg/μl)	Approach
Naphthalene	8.2	A
C1-naphthalenes	8.4	A
C2-naphthalenes	13	C
C3-naphthalenes	15	A
C4-naphthalenes	11	C
Fluorene	0.81	A
C1-fluorenes	4.2	A
C2-fluorenes	9.6	A
C3-fluorenes	44	B
Phenanthrene	4.2	A
C1-phenanthrenes & anthracenes	4.9	A
C2-phenanthrenes & anthracenes	13	A
C3-phenanthrenes & anthracenes	66	B
C4-phenanthrenes & anthracenes	120	B
Dibenzothiophene	0.18	A
C1-dibenzothiophenes	1.4	B
C2-dibenzothiophenes	1.3	A
C3-dibenzothiophenes	15	A
Fluoranthene	0.61	A
C1-fluoranthenes & pyrenes	2.5	C
C2-fluoranthenes & pyrenes	2.0	B
Chrysene & triphenylene	0.89	A
C1-Benz[a]anthracenes & chrysenes & triphenylenes	0.67	C
C2-Benz[a]anthracenes & chrysenes & triphenylenes	1.0	B

A, 1:200 replicate analysis seven times; B, 1:20 replicate analysis nine times; C, three times the background from LDPE blank analysis

The average percent recovery of the matrix positive control was 77.6% (3.51–119%) with an average relative standard deviation of 25.0% (5.52–132%). C4-phenanthrenes & anthracenes and C3-dibenzothiophenes alkylation series concentrations in the matrix spike were below instrument detection limits. Average concentrations, percent recoveries, and relative standard deviation percentages for all alkylation series can be found in ESM Table S5.

Results from the cross-check evaluation with the in-house standard were beneficial for identification of compound interferences that would affect percent recoveries for each alkylation series. The average percent recovery of the in-house standard was 93.6% (70.6–122%). 7,12-Dimethylbenz[a]anthracene was not detected as part of the C2-benz[a]anthracenes & chrysenes & triphenylenes alkylation series because it was outside the designated retention region. 9,10-Dimethylanthracene was not detected as part of the C2-phenanthrenes & anthracenes alkylation series because it was outside the designated retention region. Three alkylation series, C4-naphthalenes, C2-phenanthrenes & anthracenes, and C1-fluoranthenes & pyrenes, had percent recoveries well above 100%, indicating the presence of interferences. These three alkylation series were excluded from the average percent recovery. Interferences were identified for C1-fluoranthenes & pyrenes (see Table 5). Using full scan analysis, no interfering ion peaks were identified for C4-naphthalenes and C2-phenanthrenes & anthracenes. Therefore, these high recoveries are likely due to relative response differences between the alkylation series present in SRM 1991 compared to individual alkylated PAH standards at a single concentration in the in-house standard (discussed further in the “Method validation” section).

**Table 5:** In-house 62 PAH standard analysis using the alkylated PAH method

Analyte	Alkylation series	% recovery	Interferences
Naphthalene	Naphthalene	102%	N/A
2-Methylnaphthalene 1-Methylnaphthalene	C1-naphthalenes	76.7%	N/A
2-Ethylnaphthalene 2,6-Dimethylnaphthalene 1,6-Dimethylnaphthalene 1,3-Dimethylnaphthalene 1,4-Dimethylnaphthalene 1,5-Dimethylnaphthalene 1,2-Dimethylnaphthalene 1,8-Dimethylnaphthalene	C2-naphthalenes	108%	N/A
N/A	C3-naphthalenes		2,6-Diethylnaphthalene
2,6-Diethylnaphthalene	C4-naphthalenes	**	SRM 1991 response differences
Fluorene	Fluorene	88%	N/A
N/A	C1-fluorenes		Chromatographic noise
N/A	C2-fluorenes		Chromatographic noise
N/A	C3-fluorenes		Chromatographic noise
Dibenzothiophene	Dibenzothiophene	122%	N/A
N/A	C1-dibenzothiophenes		Chromatographic noise
N/A	C2-dibenzothiophenes	N/A	N/A
N/A	C3-dibenzothiophenes	N/A	N/A
Phenanthrene	Phenanthrene	96.4%	N/A
2-Methylphenanthrene 1-Methylphenanthrene 2-Methylanthracene 9-Methylanthracene	C1-phenanthrenes & anthracenes	83.2%	N/A
3,6-Dimethylphenanthrene 2,3-Dimethylanthracene 9,10-Dimethylanthracene	C2-phenanthrenes & anthracenes	**	9,10-Dimethylanthracene outside designated retention region, SRM 1991 response differences
N/A	C3-phenanthrenes & anthracenes		Pyrene
N/A	C4-phenanthrenes & anthracenes		Pyrene
Fluoranthene	Fluoranthene	70.6%	N/A
1-Methylpyrene	C1-fluoranthenes & pyrenes	**	Benzo(a)fluorene, Benzo(b)fluorene, Benzo(c)fluorene
N/A	C2-fluoranthenes & pyrenes		Chromatographic noise
Chrysene & triphenylene	Chrysene & triphenylene	90.4%	N/A
5-Methylchrysene 6-Methylchrysene	C1-benz[a]anthracenes & chrysenes & triphenylenes	88.8%	N/A
7,12-Dimethylbenz[a]anthracene	C2-benz[a]anthracenes & chrysenes & triphenylenes	Not detected	Outside designated retention region

*N/A* not applicable

**Interferences present, percent recovery not able to be quantified

The in-house standard did not contain alkylated PAHs from ten of the alkylation series in the method. However, all but two of these alkylation series had quantifiable detections. Interferences were identified for C3-naphthalenes, C3-phenanthrenes & anthracenes, and C4-phenanthrenes & anthracenes (see Table 5). The concentrations obtained for the remaining eight alkylation series were relatively low (all less than 22 pg/μl), and were determined to be chromatographic noise using full scan analysis.

### Standard reference materials

All known alkylation series from NIST SRMs were able to be quantitated. The average percent recovery for all SRMs, each sample’s score using our novel source ratio methodology, their known source, and their predicted source are listed in Table 6. After examining the differences in petrogenic or pyrogenic scores, the predicted source was established for each material and matched that of the known source for all five SRMs. A summary of each ratio’s success in source prediction can be found in ESM Table S29. Percent recoveries for all SRMs, their calculated ratio values, and the associated weighted scores are provided in ESM Tables S6-S15.

**Table 6:** Summary of SRM results using expanded and modified alkylated PAH method and ratio list

Sample	% recovery	Petrogenic score^a^	Pyrogenic score^a^	Score difference	Known source	Predicted source
SRM 2779	108	**14**	4	+10 petro	Petrogenic	Petrogenic
SRM 1582	98.2	**11**	3	+8 petro	Petrogenic	Petrogenic
SRM 1580	101	13	13	0 mixed	Mixed	Mixed
SRM 1597a	115	2	**22**	+20 pyro	Pyrogenic	Pyrogenic
SRM 1975	79.1	2	**12**	+10 pyro	Pyrogenic	Pyrogenic

^a^values in bold indicate the sample’s predicted source

#### SRM 2779 – Gulf of Mexico Crude Oil

The average percent recovery of SRM 2779 using the expanded and modified alkylated PAH method was 108%. The 22 ratio list predicted SRM 2779 to be petrogenic in source, with a petrogenic sum score of 14 compared to a pyrogenic sum score of 4. Eleven ratios correctly predicted a petrogenic signature. Four ratios, D2/P2, D3/P3, BeP/BaP, and PY0/BaP, incorrectly predicted SRM 2779 to be pyrogenic in source.

#### SRM 1582 – Petroleum Crude Oil

The average percent recovery of SRM 1582 using the expanded and modified alkylated PAH method was 98.2%. The 22 ratio list predicted SRM 1582 to be petrogenic in source, with a petrogenic sum score of 11 compared to a pyrogenic sum score of 3. Eight ratios correctly predicted a petrogenic signature while three ratios, FLPY/(P2+P3+P4), D2/P2, and D3/P3, incorrectly predicted SRM 1582 to be pyrogenic.

#### SRM 1580 – Organics in Shale Oil

The average percent recovery across the four analytes using the expanded and modified alkylated PAH method was 101%. In addition, we measured concentrations for all 24 alkylation series, with concentrations ranging from 1280 to 1,570,000 pg/μl. The 22 ratio list predicted SRM 1580 to be mixed in source, with a petrogenic sum score of 13 and a pyrogenic sum score of 13.

#### SRM 1597a – Complex Mixture of Polycyclic Aromatic Hydrocarbons from Coal Tar

The average percent recovery of SRM 1597a using the expanded and modified alkylated PAH method was 115%. Two alkylation series, C2-naphthalenes and C1-benz[a]anthracenes & chrysenes & triphenylenes, had high percent recoveries due to interferences, and were excluded from the average percent recovery. We also measured concentrations for eight additional alkylation series, with concentrations ranging from 530 to 313,000 pg/μl. Overall, the 22 ratio list predicted SRM 1597a to be pyrogenic in source, with a pyrogenic sum score of 22 compared to a petrogenic sum score of 2. Thirteen ratios correctly predicted a pyrogenic signature. Two of the ratios, PY0/BaP and L/H, incorrectly predicted SRM 1597a to be petrogenic in source.

#### SRM 1975 –Diesel Particulate Extract

The average percent recovery across all analytes in SRM 1975 using the expanded and modified alkylated PAH method was 79.1%. Additionally, we measured concentrations for 20 alkylation series, with concentrations ranging from 88.2 to 9790 pg/μl. The 22 ratio list predicted SRM 1975 to be pyrogenic in source, with a pyrogenic sum score of 12 compared to a petrogenic sum score of 2. Eight ratios correctly predicted a pyrogenic signature. Three ratios, BaA/Ch0, BaA/228, and L/H, incorrectly predicted SRM 1975 to be petrogenic in source.

### Known PAH source samples

A summary of each ratio’s success in source prediction for all samples can be found in ESM Table S29. Calculated ratio values and their associated weighted scores are provided in ESM Tables S23-S28.

#### Deepwater Horizon

**Fig. 2:**
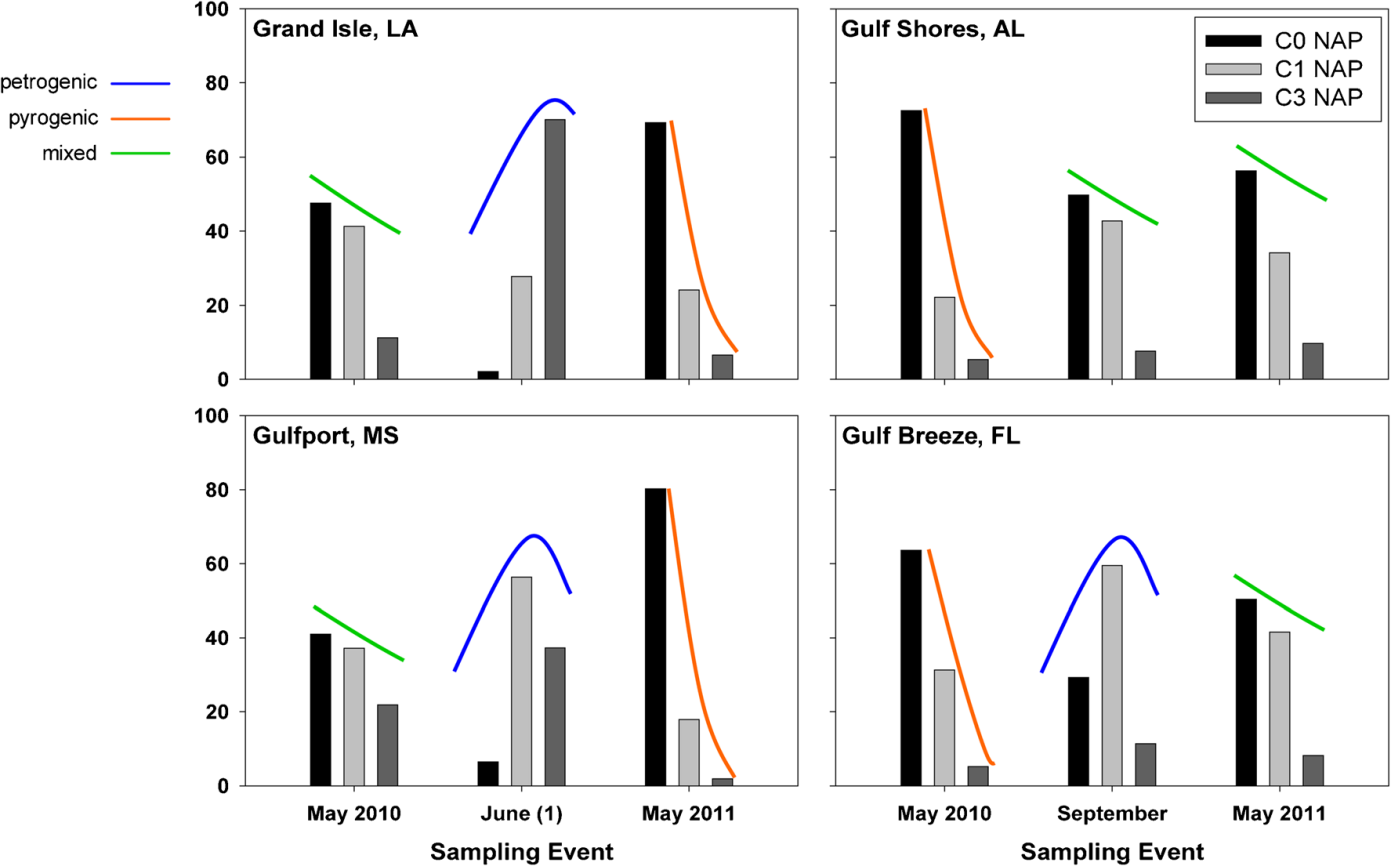
Deepwater Horizon sample pattern sourcing predictions [9]

Using a traditional approach, petrogenic signatures were previously observed in June 2010 for Grand Isle, LA, and Gulfport, MS, and in September 2010 for Gulf Breeze, FL (Fig. 2) [9].

As seen in Table 7, the samples analyzed from Grand Isle, LA, correspond strongly to the signatures observed in Fig. 2 from a previous study, with petrogenic sum scores of 24 and 25 versus pyrogenic sum scores of 3 and 3. Three ratios, FLPY/(P2+P3+P4), D2/P2, and D3/P3, incorrectly predicted Grand Isle, LA, samples to be pyrogenic in source. Samples analyzed from Gulfport, MS, and Gulf Breeze, FL, displayed a stronger pyrogenic signature than petrogenic.

**Table 7:** Samples collected along gulf coast during DWH incident with predicted source summation scores

Site location	Deployment date	Petrogenic score^a^	Pyrogenic score^a^	Score difference
Grand Isle, LA	June 2010	**24**	3	+21 petro
Grand Isle, LA	June 2010	**25**	3	+22 petro
Gulfport, MS	June 2010	9	**18**	+9 pyro
Gulfport, MS	June 2010	8	**16**	+8 pyro
Gulfport, MS	August 2010	8	**19**	+11 pyro
Gulf Breeze, FL	August 2010	8	**20**	+12 pyro

^a^values in bold indicate the sample’s predicted source

#### St. Helens, OR, creosote site

Results from the expanded and modified alkylated PAH method and ratio prediction are displayed in Table 8. The majority of the samples were predicted to be pyrogenic in nature, with a few exceptions. The air sample at site A, shallow porewater sample at site B, and deep porewater sample at C were predicted to be mixed in source.

**Table 8:** Predicted Source Summation Scores for all St. Helens Sampling Locations

	Site ID	Air^a^	Water^b^	Shallow porewater	Deep porewater
Upstream	A	Mixed	+7 pyro	+7 pyro	+5 pyro
↓	B	N/A	+4 pyro	Mixed	+3 pyro
	C	N/A	N/A	+11 pyro	Mixed
	D	N/A	N/A	+14 pyro	+11 pyro
	E	N/A	N/A	+12 pyro	+12 pyro
	F	N/A	+8 pyro	+7 pyro	+11 pyro
	G	N/A	N/A	+15 pyro	+5 pyro
	H	+7 pyro	+9 pyro	+16 pyro	+7 pyro
	I	N/A	N/A	+12 pyro	+16 pyro
	J	N/A	N/A	+13 pyro	+5 pyro
	K	N/A	+9 pyro	+12 pyro	+13 pyro
	L	N/A	N/A	+13 pyro	+15 pyro
	M	N/A	+4 pyro	+15 pyro	+11 pyro
	N	+4 pyro	+9 pyro	+3 pyro	+11 pyro
Downstream	O	N/A	+12 pyro	+10 pyro	+8 pyro

^a^*N/A*, no samples collected in air compartment at this site

^b^*N/A*, no samples collected in water compartment at this site

Nine ratios correctly predicted pyrogenic signatures in water samples. Seven ratios, PA1/PA0, PA0/PA01, PY0/BaP, ∑alkyl/parent PAHs, L/H, LPAH/HPAH, and pyrogenic index, predicted a petrogenic source. Ratio values from all water samples were then compared with reference creosote ratios [5, 6]. Five ratios, A0/PA0, P0/A0, FLP1/FLPY0, BaA/Ch0, and LPAH/HPAH, matched creosote signatures across an average of 88.0% (50.0–100%) of samples. For twelve ratios, PA1/PA0, FL0/PY0, FL0/FLPY, FLPY/(P2 + P3 + P4), BaA/228, D2/P2, D3/P3, IP/ghi, IP/IP + ghi, BeP/BaP, L/H, and pyrogenic index, reference creosote values did not match any water sample ratio values. Five ratios, PA0/PA01, FLPY0/FLPY01, FLP1/PY0, PY0/BaP, and ∑alkyl/PAHs, did not have literature ratio values for creosote sources.

Eleven ratios correctly predicted porewater samples to be pyrogenic in source. Five ratios, PY0/BaP, ∑alkyl/parent PAHs, L/H, LPAH/HPAH, and pyrogenic index, were found to incorrectly predict a petrogenic source. These five ratios were also found to incorrectly predict petrogenic signatures in water. Ratio values from all pyrogenic porewater samples were also compared with reference creosote ratios [5, 6]. Ten ratios, A0/PA0, P0/A0, PA1/PA0, FL0/PY0, FL0/FLPY, FLP1/FLPY0, FLPY/(P2 + P3 + P4), BaA/Ch0, D2/P2, and IP/ghi, matched creosote signatures across an average of 53.1% (9.09–100%) of porewater samples. For nine ratios, PA1/PA0, FL0/FLPY, BaA/228, D3/P3, IP/IP + ghi, BeP/BaP, L/H, LPAH/HPAH, and pyrogenic index, reference creosote values did not match any porewater sample ratio values.

#### Fire chamber study

Results from the expanded and modified alkylated PAH method and ratio prediction are displayed in Table 9. Both chamber study samples were predicted to be strongly pyrogenic in source, with sum scores of 21 and 19. Seventeen ratios correctly predicted pyrogenic source. Three ratios, L/H, LPAH/HPAH, and pyrogenic index, incorrectly predicted petrogenic source. In both samples, C2-dibenzothiophenes, C2-phenanthrenes & anthracenes, C3-dibenzothiophenes, C3-phenanthrenes & anthracenes, and C4-phenanthrenes & anthracenes were not detected.

**Table 9:** Predicted source summation scores for fire chamber samples

	Petrogenic value	Pyrogenic value^a^	Score difference	Known source	Predicted source
Sample 1	5	**19**	+14 pyro	Pyrogenic	Pyrogenic
Sample 2	2	**21**	+19 pyro	Pyrogenic	Pyrogenic

^a^values in bold indicate the sample’s predicted source

### Forensic ratios

Of the 22 ratios used, 60% were successful predictors of all SRMs and environmental samples. Based on the results, nine ratios incorrectly predicted PAH sources: D2/P2, D3/P3, PY0/BaP, IP/ghi, IP/IP+ghi, BeP/BaP, L/H, LPAH/HPAH, and pyrogenic index. These nine unsuccessful ratios were removed from the list, leaving thirteen top performing ratios (see Table 10). Using the same scoring criteria outlined in the “Forensic ratios” section, all sample ratio values and weighted scores were recalculated. All source predictions remained the same with the exception of SRM 1580. With a new petrogenic score of 12 and a pyrogenic score of 9, SRM 1580 (Shale Oil) was predicted to be petrogenic in source.

**Table 10:** Top thirteen forensic sourcing ratios with petrogenic and pyrogenic categories and associated weighted score

Abbreviation^a^	Ratio	Pyro	Petro	Pyro score	Petro score	Ref^b^	PAH ratio category
**A0/PA0**	A / A + P	> 0.1	< 0.1	3	3	2, 3	Low molecular weight PAHs
**P0/A0**	P / A	< 5	> 30	3	3	1	Low molecular weight PAHs
**PA1/PA0**	C1 P&A / P + A	< 1	> 1.5	1	2	1	Low molecular weight PAHs
PA0/PA01	P + A / P + A + C1 P&A	> 0.5	≤ 0.4	1	1	2	Low molecular weight PAHs
FL0/PY0	FL / PY	> 1	≤ 0.5	1	1	1	4-ringed PAHs
FL0/FLPY	FL / FL + PY	> 0.5	< 0.4	1	2	2, 3	4-ringed PAHs
**FLPY0/FLPY01**	FL + PY / FL + PY + C1 FL&PY	> 0.5	< 0.5	3	3	2	4-ringed PAHs
FLP1/PY0	C1 FL&PY / PY	~ 0.3	~ 4	1	1	1	4-ringed PAHs
FLP1/FLPY0	C1 FL&PY / FL + PY	< 1	> 1	2	1	1	4-ringed PAHs
FLPY/(P2 + P3 + P4)	FL + PY / (C2 + C3 + C4) P&A	< 0.3	> 9	1	1	1	4-ringed PAHs
BaA/Ch0	BaA / Ch	≥ 0.5	< 0.25	1	1	1	4-ringed PAHs
BaA/228	BaA / BaA + Ch + Trp	> 0.35	< 0.2	1	1	2, 3	4-ringed PAHs
∑alkyl/PAHs	(C1 + C2 + C3 + C4) Nap + (C1 +C2 +C3) P&A + (C1 +C2) FL&PY + C1 BaA&Ch&Trp / Nap + P + A + FL + PY + BaA + Ch	< 1	> 2.3	2	1	1	Alkyl/parent PAHs

^a^Abbreviations in bold indicate the ratio is definitive of a source

^b^1. Stogiannidis, E.; Laane, R.; Source characterization of polycyclic aromatic hydrocarbons by using their molecular indices: an overview of possibilities. In *Review of Environmental Contamination and Toxicology*; Whitacre, D.M., Ed.; Springer: New York, NY, 2014; 234, 49–177

2. Yunker, M.B.; Macdonald, R.W.; Vingarzan, R.; Mitchell, R.H.; Goyette, D.; Sylvestre, S.; PAHs in the Fraser River Basin: a critical appraisal of PAH ratios as indicators of PAH source and composition. *Org Geochem* 2002; 33, 489–515

3. Tobiszewski, M.; Namiesnik, J. PAH diagnostic ratios for the identification of pollution emission sources. *Environ Pollution* 2012; 162, 110–119

## Discussion

### Method validation

The calibration range for our expanded, modified method is 0.0017 to 28 ng/μl based on C2-naphthalenes with resulting coefficients of determination (*r*^2^) > 0.99. The calibration range is two orders of magnitude larger than that used in ASTM D7363-13 (0.0017 ng/μl to 0.34 ng/μl), which facilitates faster analysis by minimizing dilutions. The expanded, modified method also includes six analytes/alkylation series not present in the original ASTM method: dibenzothiophene, C1-dibenzothiophenes, C2-dibenzothiophenes, C3-dibenzothiophenes, C2-fluoranthenes & pyrenes, and C2-benz[a]anthracenes & chrysenes & triphenylenes.

We had good recovery with inter- and intra-day laboratory analysis (ESM Table S4) and the matrix positive control. We also had good recovery with the in-house PAH standard, and could identify all interferences. Low-level detections for five alkylation series were attributed to chromatographic noise and were background subtracted. For four alkylation series, interferences from other PAH compounds in the in-house standard led to high percent recoveries and also could be background subtracted. Two additional alkylation series, C4-naphthalenes and C2-phenanthrenes & anthracenes, had high percent recoveries due to response differences between SRM 1991 and the in-house standard. However, since most alkylated PAH standards are not available, alternative approaches need to be used. ASTM D7363-13a [7] was established using NIST SRM 1991, which is an environmental matrix consisting of mixed coal tar/petroleum non-aqueous phase liquid (NAPL) that has been diluted in methylene chloride. Using a real-world, oil-based standard introduces matrix effects and contains chemicals that have not yet been identified. Therefore, by using an SRM to calibrate the method, we may not account for all chemicals in a given alkylation series. This situation likely explains why 7,12-dimethylbenz[a]anthracene was outside the designated integration even though it is classified as a C2-benz[a]anthracene & chrysene & triphenylene. A potential future direction is to create a stock solution containing multiple alkylated PAH SRMs to try and account for additional compounds. By including multiple SRMs from different sources, we would increase the likelihood of capturing more alkylated PAHs than those present in just SRM 1991.

### Standard reference materials

The average percent recovery across all SRMs, after accounting for known interferences, was 100%. Like SRM 1991, the SRMs tested for method validation are real-world standards with their own matrix that could affect percent recoveries. Additional alkylated PAHs could also be present in the samples that were not included on the certificate of analysis. Our modified, expanded alkyl PAH method would detect these compounds in the alkylation series integration, resulting in a higher percent recovery of the alkylation series.

Higher percent recoveries in all SRMs may be due to method calibration with an SRM rather than individual standards. Each SRM will have a unique combination of alkylated PAHs based on the formation of the material and weathering over time. This difference will lead to varying response factors at the instrument for each alkylation series. Therefore, the calculated concentration of SRMs in comparison to the calibration solution SRM 1991 will have more uncertainty. In addition, NIST reference mass fraction values have a larger uncertainty range than certified mass fraction values which could lead to more error in the calculated percent recovery.

### Known PAH source samples

Using our new analytical method and list of 22 ratios, two of the six of the water samples analyzed from the gulf coast during the Deepwater Horizon incident were predicted to be petrogenic in signature. These results correspond to the geographic proximity of Grand Isle, LA, which was located closest to the spill and had little physical protection from the Gulf of Mexico current trajectory during the oil spill [12]. A possible explanation for the pyrogenic signatures in the Mississippi and Florida samples is that these sites are further from the incident, had less direct impact from the spill over the sampling duration, and are closer to urban centers. Gulfport, MS, was protected by offshore barrier islands and Gulf Breeze, FL, was protected by the natural peninsula and a boom put in place for additional protection [12]. In addition, both sites were impacted to some degree by urban and industrial activities, and it could be that these additional PAH sources dominated those of the oil spill. Another contributing factor could be the use of in-situ burning and dispersants as remediation attempts, which could have changed the composition of the oil, leading to a different signature. These results highlight the importance of using multiple ratios to effectively analyze potential sources of PAH contamination in the environment.

The St. Helens, OR, former creosote site is subject to heavy boat traffic, other industrial processes, and a set of in-use train tracks that run across the northern side of the property. While sampling, a wildfire occurred 121,000 m southeast of the site, and spread westward to as close as 88,500 m away [13]. These sources all contribute pyrogenic PAHs to the air, and would be captured by the LDPE air samples. These PAH inputs would lead to different predictions of sourcing between water and air matrices depending on season and surrounding conditions. The complex contribution of pyrogenic sources makes it difficult to isolate creosote contamination as the dominate source of PAHs in air, but helps explain why our ratios reported a score difference of 4–7 pyrogenic (see Table 8).

Pyrogenic signatures were observed across all St. Helens water samples, likely indicative of creosote contamination, but could have been mixed with pyrogenic sources like wildfires as mentioned above. Two of the five ratios matching creosote signatures in samples were given weighted scores of 3 and considered to be definitive of a source. All porewater samples were predicted to be pyrogenic in source, except for site B shallow porewater and site C deep porewater. Three of the ten ratios matching creosote signatures in samples were given weighted scores of 3 and considered to be definitive of a source. Because this location has been exposed to many legacy and modern day sources of PAHs, we cannot completely eliminate petrogenic signatures from these samples.

Our two samples from the fire chamber study were both predicted to be strongly pyrogenic. In this study, passive samplers were exposed to a high degree of combustion and fire source in close proximity within a closed space. The results of a strong pyrogenic signature (score differences of +14–19 pyrogenic, see Table 9) therefore correspond to the design of the study, and helped to validate the alkylated ratio scoring system.

### Forensic ratios

SRM 1580, a shale oil standard, was predicted to have a mixed PAH signature based on ratios and weighted scores. Shale oil originates from shale rock, and is obtained through oil extraction from within the layers of rock formations using a pyrolysis process. This process can either consist of using heat to release the oil from the rock, or in-situ extraction in which the rock is heated underground and the resulting gas vapors are condensed and pumped out [14]. This process of high heat could lead to the introduction of pyrogenic signatures, causing the oil to be less petrogenic in signature. Despite PAH mixing, the original source material is based on a petrogenic oil. The refined top 13 performing ratios were sensitive enough to detect the original source, and scored SRM 1580 as +3 petrogenic.

Overall, the applied weighted scores were supported by the source predictions of SRMs and environmental samples. Ratios given a weighted score of 3 always correctly predicted sample source. Ratios with a value of 2 that were used in the literature to corroborate other ratios matched the predictions of more definitive ratios. Ratios that were not well studied, sensitive to weathering or unreliable and given a weighted score of 1 or 2 could not distinguish between petrogenic and pyrogenic sources or incorrectly predicted a sample’s source. By assigning weighted scores to ratios, the sum score for all SRMs and environmental samples correctly predicted the PAH source.

Based on our results, we found 13 ratios were effective in predicting PAH sources in at least 90% of samples. Low molecular weight and 4-ringed PAH ratios appeared to be the most successful predictors of PAH sources. The results are similar to other PAH ratio reviews [4–6]. Using only the top performing 13 ratios simplifies both the analytical and computational effort, yet still provides accurate predictions for the SRMs and environmental samples tested.

For each forensic PAH ratio, specific materials such as crude oil, diesel, wood combustion, and creosote have been tested within each general source category of petrogenic and pyrogenic. A potential future direction of this study is to subcategorize petrogenic and pyrogenic ratio values into ranges for potential source materials. Weighted scores could also be subcategorized for source materials that have been more extensively tested, are more resistant to weathering, and more definitive based on the ratio being tested. Refining ratio values would allow us to further distinguish PAH sources in samples, helping to identify exposure routes of PAH contamination.

## Conclusion

After expanding and modifying the ASTM D7363-13a [7] method to a gas chromatography triple quadrupole mass spectrometry instrument, we obtained a calibration range that was two orders of magnitude larger and contained an additional six analytes/alkylation series. To the authors’ knowledge, these are the lowest reported instrument LOQs for alkylated PAH series. Using an in-house standard containing parent and alkylated PAHs, interferences in alkylation series were identified. The use of an SRM for a calibration solution introduces matrix effects and likely contains compounds that have not yet been characterized. However, making a calibration solution containing all alkylated PAH compounds would be unrealistic due to cost and standard availability. A potential future direction is to create a stock solution containing multiple alkylated PAH SRMs to try and account for additional compounds.

Using our expanded, modified alkyl PAH analytical method and the combined list of 22 ratios, we were able to accurately predict the source of all standards and environmental samples. These results highlight the strengths of a converging lines of evidence approach using multiple sourcing ratios and weighted scores. In addition, we identified weaknesses in individual ratios within the compiled list, and created a refined list of the thirteen most robust ratios. A potential future direction is to further tease out and identify specific sources within petrogenic and pyrogenic categories. Knowing the limitations of individual ratios is still important when analyzing samples from unknown sources. Additionally, having some historical information on the sample collection site can also serve as a useful tool in appropriately applying and interpreting ratio results.

## Supplementary information

ESM 1 (PDF 2.23 mb)

ESM 2 (XLSX 286 kb)

## Data Availability

Data is available in supplemental information, and an Excel version of the ratio list is provided.
