# Supplementary material for: Improvements in identification and quantitation of alkylated PAHs and forensic ratio sourcing
Source: Anal Bioanal Chem. 2021 Jan 28;413(6):1651–64. doi: 10.1007/s00216-020-03127-0 (PMC7921031; doi:10.1007/s00216-020-03127-0)
Supplement: Supplementary file 1 — (PDF 2.23 mb) [file 216_2020_3127_MOESM1_ESM.pdf]

Improvements in identification and quantitation of alkylated PAHs and forensic ratio sourcing

Analytical and Bioanalytical Chemistry

Christine C. Ghetu[1], Richard P. Scott[1], Glenn Wilson[1], Rachel Liu-May[2], Kim Anderson*[1]

[1]Food Safety and Environmental Stewardship Program, Department of Environmental and Molecular Toxicology, Oregon State University, 1007 Ag. and Life Sciences Building, Corvallis, Oregon, USA
[2] Department of Chemistry, Oregon State University, Corvallis, OR 97331, USA
* corresponding author kim.anderson@oregonstate.edu

Supplemental Information

Table	Page #
**Table S1**. Alkylated PAH Physicochemical Properties	1
**Table S2**. Instrument Parameters	2
**Table S3**. Analyte Retention Ranges	6
**Table S4**. Inter-day and Intra-day Study Results	7
**Table S5**. Low Density Polyethylene Matrix Spike with SRM 1991	8
**Table S6**. SRM 2779 Percent Recovery	9
**Table S7**. SRM 2779 Ratio Results	10
**Table S8**. SRM 1582 Percent Recovery	11
**Table S9**. SRM 1582 Ratio Results	12
**Table S10**. SRM 1580 Percent Recovery	13
**Table S11**. SRM 1580 Ratio Results	14
**Table S12**. SRM 1597a Percent Recovery	15
**Table S13**. SRM 1597a Ratio Results	16
**Table S14**. SRM 1975 Percent Recovery	17
**Table S15**. SRM 1975 Ratio Results	18
**Table S16**. SRM Instrument Concentrations	19-20
**Table S17**. Deepwater Horizon Environmental Water Concentrations	20-21
**Table S18**. St Helens Air Concentrations	22
**Table S19**. St Helens Environmental Water Concentrations	23-24
**Table S20**. St Helens Environmental Shallow Porewater Concentrations	24-25
**Table S21**. St Helens Environmental Deep Porewater Concentrations	26-27
**Table S22**. Chamber Study Instrument Concentrations	28
**Table S23**. Deepwater Horizon Ratio Results	29
**Table S24**. St Helens Air Ratio Results	30
**Table S25**. St Helens Water Ratio Results	31
**Table S26**. St Helens Shallow Porewater Ratio Results	32
**Table S27**. St Helens Deep Porewater Ratio Results	33
**Table S28**. Chamber Study Ratio Results	34
**Table S29**. PAH Ratio Accuracy in Source Prediction for SRMs and Known PAH Source Samples	35

Figure	Page #
**Figure S1.** Naphthalene Series Peak Integration	3
**Figure S2.** Fluorene Series Peak Integration	3
**Figure S3.** Phenanthrene & anthracene Series Peak Integration	4
**Figure S4.** Dibenzothiophene Series Peak Integration	4
**Figure S5.** Fluoranthene & pyrene Series Peak Integration	5
**Figure S6.** Benz[a]anthracenes & chrysenes & triphenylenes Series Peak Integration	5

Table S1. Alkylated PAH Physicochemical Properties

Analyte	CAS	MW (g mol^{-1})	log K_{ow}[a]	log K_{oa}[b]	H_{298} (atm m^3 mol^{-1})[c]
naphthalene	91-20-3	128.2	3.3	5.05	$5.26x10^{-4}$
C1-naphthalenes		142.2	3.3	5.05	$5.80x10^{-4}$
C2-naphthalenes		156.2	3.3	5.05	$6.41x10^{-4}$
C3-naphthalenes		170.2	3.3	5.05	$6.41x10^{-4}$
C4-naphthalenes		184.3	4.18	5.05	$1.13x10^{-3}$
fluorene	86-73-7	166.2	4.18	6.59	$1.67x10^{-4}$
C1-fluorenes		180.2	4.18	6.59	$1.67x10^{-4}$
C2-fluorenes		194.3	4.18	6.59	$1.67x10^{-4}$
C3-fluorenes		208.3	4.18	6.59	$1.67x10^{-4}$
phenanthrene	85-01-8	178.2	4.46	7.22	$5.13x10^{-5}$
C1-phenanthrenes & anthracenes		193.3	4.46	7.22	$5.13x10^{-5}$
C2-phenanthrenes & anthracenes		208.3	4.46	7.22	$5.13x10^{-5}$
C3-phenanthrenes & anthracenes		223.3	4.46	7.22	$5.13x10^{-5}$
C4-phenanthrenes & anthracenes		238.4	4.46	7.22	$5.13x10^{-5}$
dibenzothiophene	132-65-0	184.3	7.24	7.24	$2.79x10^{-5}$
C1-dibenzothiophenes		199.3	7.24	7.24	$2.79x10^{-5}$
C2-dibenzothiophenes		214.3	7.24	7.24	$2.79x10^{-5}$
C3-dibenzothiophenes		228.4	7.24	7.24	$2.79x10^{-5}$
fluoranthene	206-44-0	202.3	5.16	8.60	$8.30x10^{-6}$
C1-fluoranthenes & pyrenes		217.3	5.16	8.60	$8.30x10^{-6}$
C2-fluoranthenes & pyrenes		232.3	5.16	8.60	$8.30x10^{-6}$
chrysene & triphenylene	218-01-9<br>217-59-4	228.3	5.81	9.48	$5.01x10^{-6}$
C1-benz[a]anthracenes & chrysenes & triphenylenes		243.3	5.81	9.48	$5.01x10^{-6}$
C2-benz[a]anthracenes & chrysenes & triphenylenes		258.3	5.81	9.48	$5.01x10^{-6}$

[a] *Experimental values reported in [1]*
Values for alkyl homologues are taken from the parent PAH
[b] *All values are KOAWIN v1.10 [1]*
Values for alkyl homologues are taken from the parent PAH
[c] *Henry's law constant at 298: estimated via the bond method for the parent PAH [1]*
Values for alkyl homologues are taken from the parent PAH

Table S2. Instrument Parameters

**Carrier Gas**			
Helium (99.99%)			
**Oven Program**		**Inlet Settings**	
hold 60°C for 1 min<br>ramp 40°C/min to 180°C<br>ramp 3°C/min to 230°C<br>ramp 1.5°C/min to 235°C<br>ramp 15°C/min to 280°C, hold for 10min<br>ramp 6°C/min to 298°C<br>ramp 16°C/min to 350°C, hold 4min<br>Total run time: 47.25 min		**Mode**	Pulsed Splitless
		**Injection Temperature (°C)**	320
		**Pulse Pressure**	35 psi Until 0.3 min
		**Purge Flow**	25 mL/min at 0.7 min
**Column Settings & Specifications**		**MSD Settings**	
J&W Select PAH, Part # CP7462, L 30 m, ID 0.25 mm, Film 0.15 µm		**MSD Transfer Line Temp**	320°C
		**Source Temp**	340°C
**Mode**	Constant Flow	**Collision Gas (purity)**	Nitrogen (99.99%)
**Flow Rate**	2 mL/min	**Flow Rate**	1.5 mL/min

Analyte Quantifier Ions and Collision Cell Voltages

**Analyte**	**Quantifier Precursor Ion**	**Quantifier Product Ion**	**Collision Energy (V)**
naphthalene-d_8	136	108	20
naphthalene	128	102	20
C1-naphthalenes	142	141	20
C2-naphthalenes	156	141	20
C3-naphthalenes	170	155	20
C4-naphthalenes	184	169	20
acenaphthylene-d_8	160	158	30
fluorene	166	165	20
C1-fluorenes	180	165	20
C2-fluorenes	194	179	20
C3-fluorenes	208	193	20
phenanthrene-d_{10}	188	160	30
phenanthrene	178	152	25
C1-phenanthrenes & anthracenes	192	191	20
C2-phenanthrenes & anthracenes	206	191	20
C3-phenanthrenes & anthracenes	220	205	20
C4-phenanthrenes & anthracenes	234	219	20
phenanthrene-d_{10}	188	160	30
dibenzothiophene	184	152	30
C1-dibenzothiophenes	198	197	5
C2-dibenzothiophenes	212	211	5
C3-dibenzothiophenes	226	225	5
fluoranthene-d_{10}	212	208	35
fluoranthene	202	200	35
C1-fluoranthenes & pyrenes	216	215	30
C2-fluoranthenes & pyrenes	230	215	20
chrysene-d_{12}	240	236	40
chrysene & triphenylene	228	226	40
C1-benz[a]anthracenes & chrysenes & triphenylenes	242	241	20
C2-benz[a]anthracenes & chrysenes & triphenylenes	256	241	20

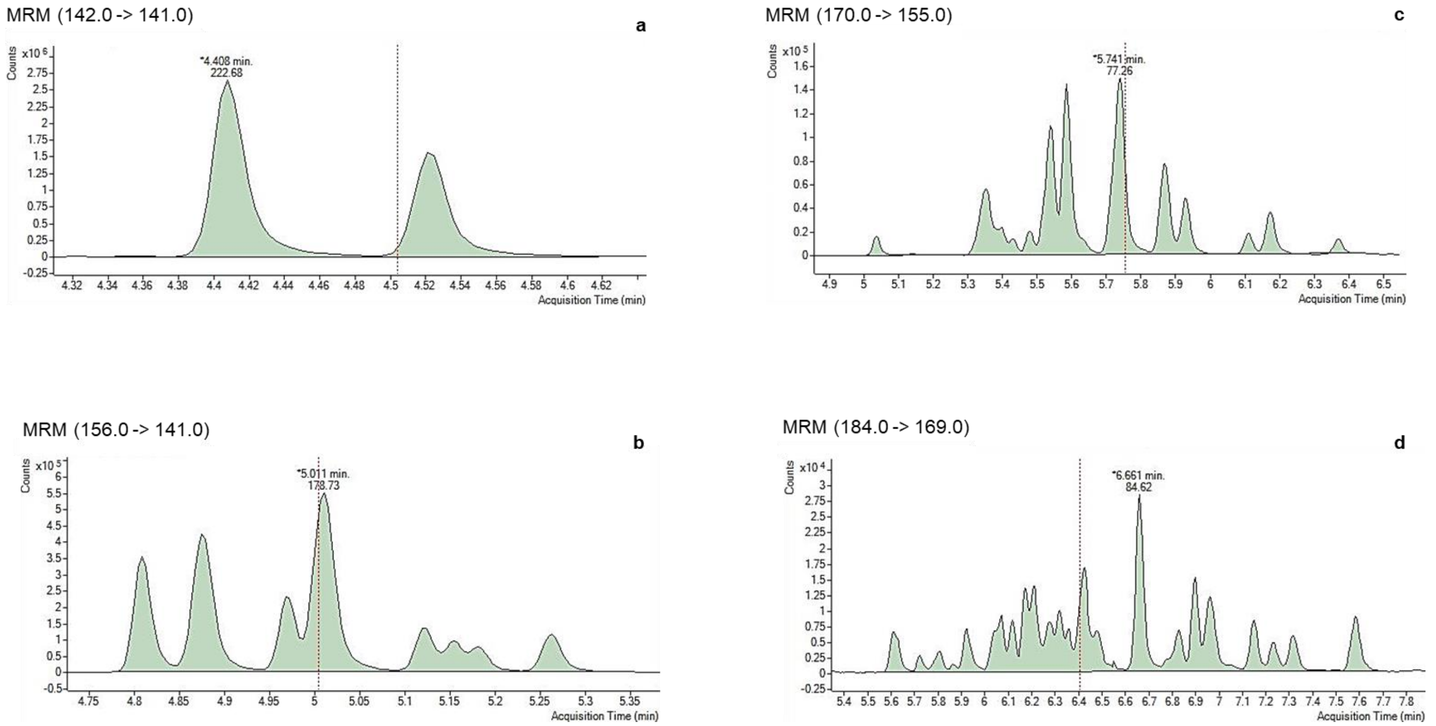

Figure S1. Naphthalene Series Peak Integration
a: C1-naphthalenes **b:** C2-naphthalenes **c:** C3-naphthalenes **d:** C4-naphthalenes

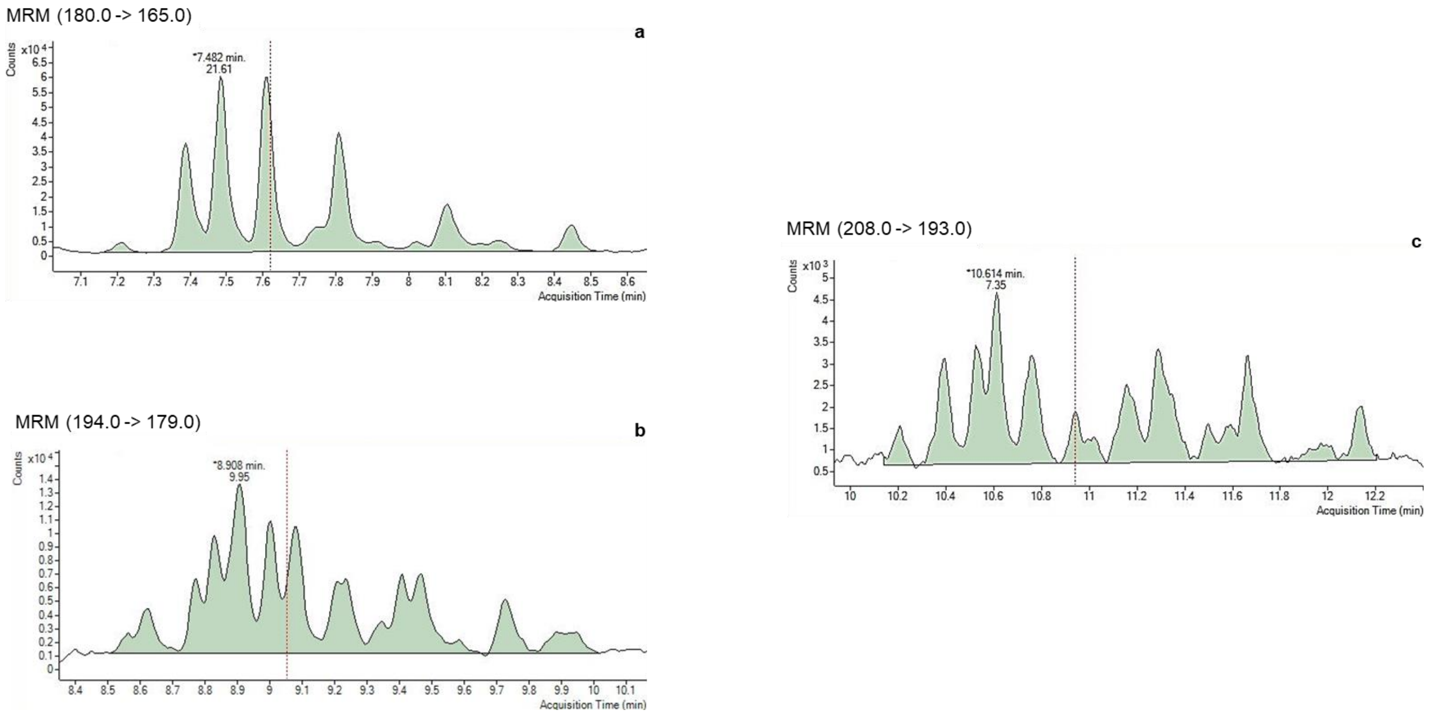

Figure S2. Fluorene Series Peak Integration
a: C1-fluorenes **b:** C2-fluorenes **c:** C3-fluorenes

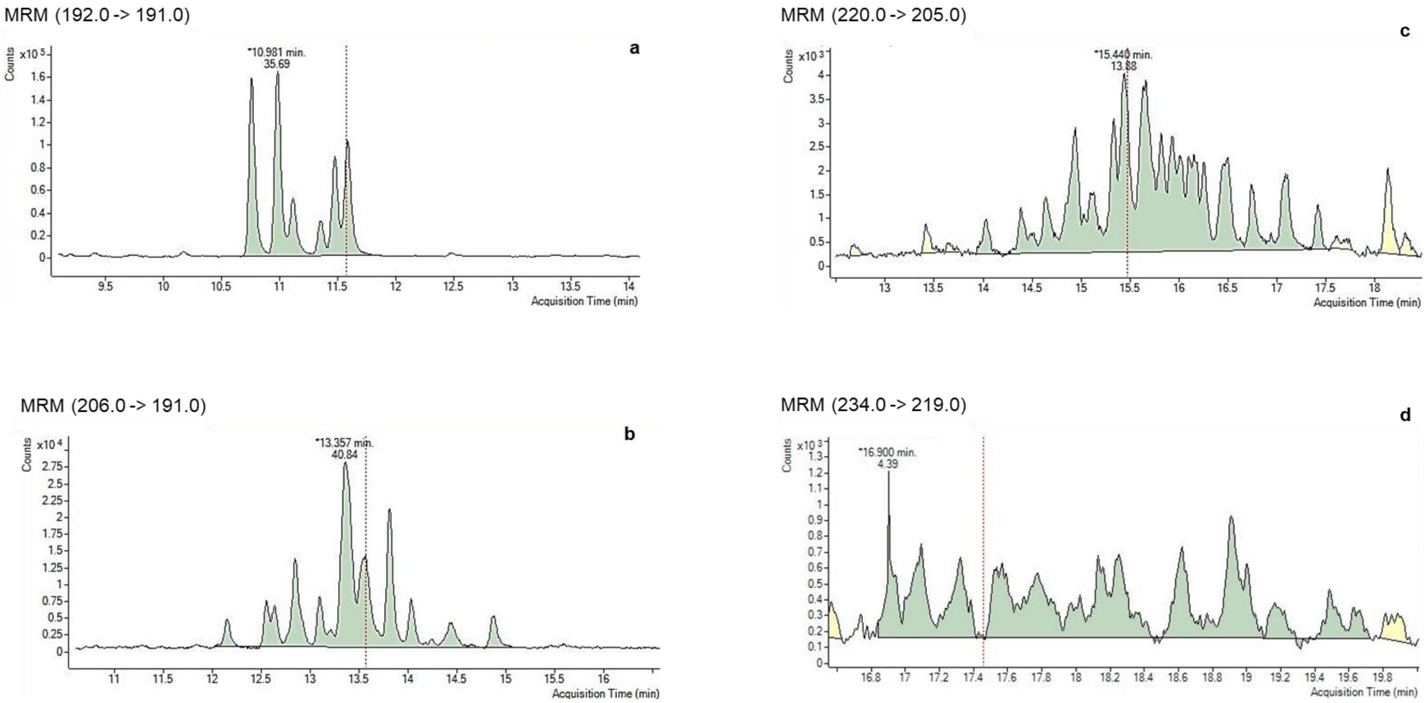

Figure S3. Phenanthrene & anthracene Series Peak Integration
a: C1-phenanthrenes & anthracenes **b:** C2-phenanthrenes & anthracenes **c:** C3-phenanthrenes & anthracenes **d:** C4-phenanthrenes & anthracenes

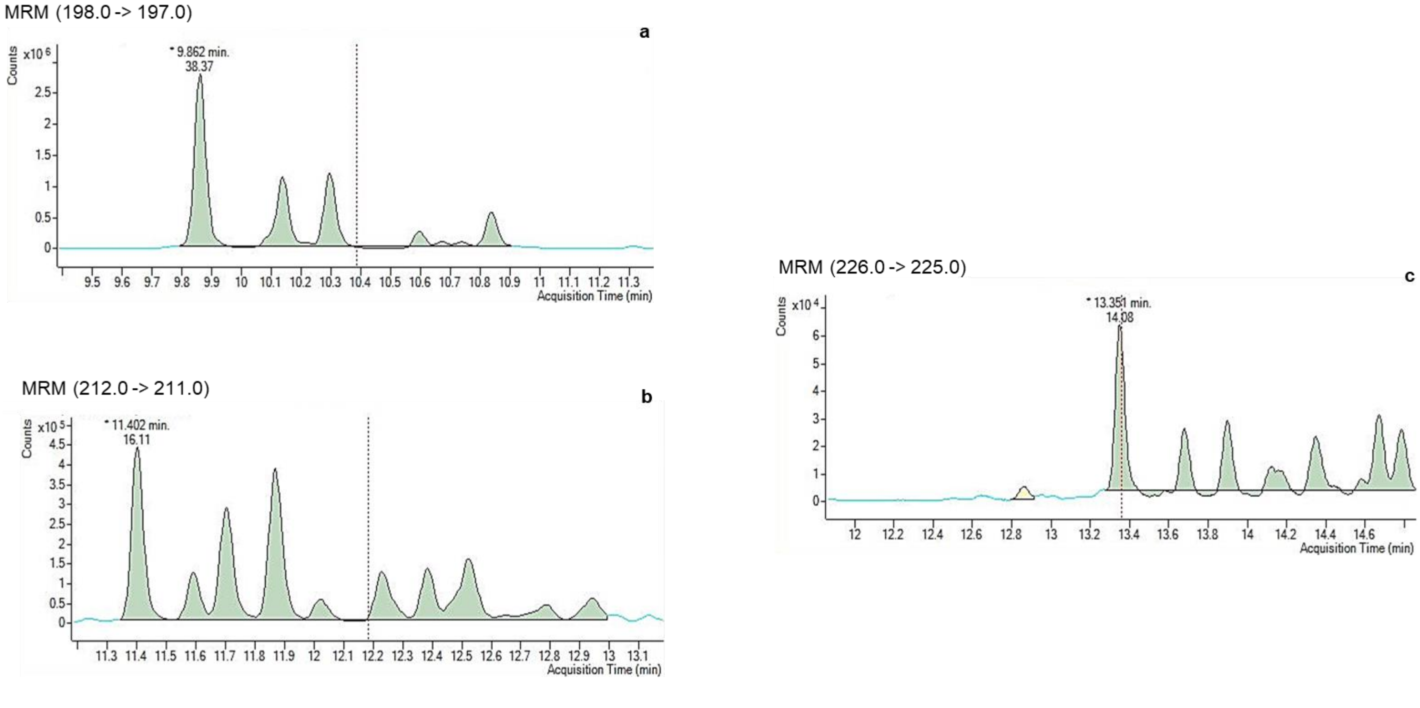

Figure S4. Dibenzothiophene Series Peak Integration
a: C1-dibenzothiophenes **b:** C2-dibenzothiophenes **c:** C3-dibenzothiophenes

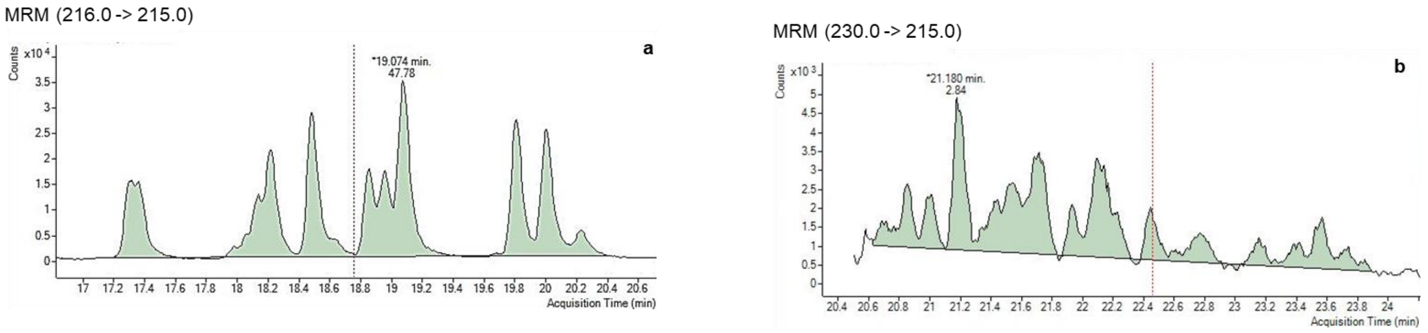

Figure S5. Fluoranthene & pyrene Series Peak Integration
a: C1-fluoranthenes & pyrenes **b:** C2-fluoranthenes & pyrenes

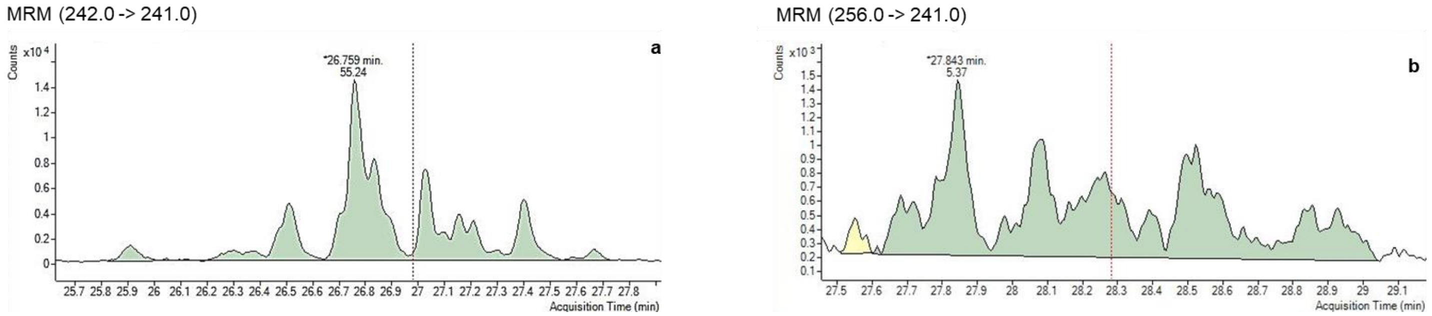

Figure S6. Benz[a]anthracenes & chrysenes & triphenylenes Series Peak Integration
a: C1-benz[a]anthracenes & chrysenes & triphenylenes **b:** C2-benz[a]anthracenes & chrysenes & triphenylenes

Table S3. Analyte Retention Ranges

Analyte[a]	Type	Integration Range (min)	Number of Calibration Points
naphthalene-d_{8}*	Internal Standard	3.96	N/A
naphthalene	Group Reference	3.98	7
C1-naphthalenes	Target	4.37 - 4.59	7
C2-naphthalenes	Target	4.76 - 5.33	7
C3-naphthalenes	Target	5.00 - 6.41	7
C4-naphthalenes	Target	5.55 - 7.68	7
acenaphthylene-d_{8}*	Internal Standard	5.42	7
fluorene	Group Reference	6.40	7
C1-fluorenes	Target	7.16 - 8.56	7
C2-fluorenes	Target	8.49 - 10.00	5
C3-fluorenes	Target	10.2 - 12.2	5
phenanthrene-d_{10}*	Internal Standard	9.09	N/A
phenanthrene	Group Reference	9.18	7
C1-phenanthrenes & anthracenes	Target	10.7 - 11.8	7
C2-phenanthrenes & anthracenes	Target	12.1 - 15.1	7
C3-phenanthrenes & anthracenes	Target	13.9 - 17.5	5
C4-phenanthrenes & anthracenes	Target	16.9 - 19.6	4
phenanthrene-d_{10}*	Internal Standard	9.09	N/A
dibenzothiophene	Group Reference	8.57	7
C1-dibenzothiophenes	Target	9.88-10.9	6
C2-dibenzothiophenes	Target	11.4-13.0	4
C3-dibenzothiophenes	Target	13.3-14.9	3
fluoranthene-d_{10}*	Internal Standard	14.8	N/A
fluoranthene	Group Reference	14.9	7
C1-fluoranthenes & pyrenes	Target	17.2 - 20.4	7
C2-fluoranthenes & pyrenes	Target	20.6 - 23.8	4
chrysene-d_{12}*	Internal Standard	25.3	N/A
chrysene & triphenylene	Group Reference	25.5	7
C1- benz[a]anthracenes & chrysenes & triphenylenes	Target	25.8 - 27.5	6
C2- benz[a]anthracenes & chrysenes & triphenylenes	Target	27.6 - 29.0	3
perylene-d_{12}*	Instrument Internal Standard	31.8	N/A

[a]*LOQs for internal standards indicated by a * are obtained from the 63 PAH method[2]*
[b]*Limits of detection (LOQ) were calculated using a 1:200 dilution of SRM 1991 analyzed 7 times or a 1:20 dilution of SRM 1991 analyzed 9 times across three different days (indicated by ^) by the student T value. Values in bold were calculated using a matrix blank.*

Table S4. Inter-day and Intra-day Study Results

Analysis Date	Inter-day Study			Intra-day Study		
**Analyte Name**	**Average (pg/µL)**	**% Recovery**	**% RSD**	**Average (pg/µL)**	**% Recovery**	**% RSD**
naphthalene-d_8	Internal Standard					
naphthalene	180	106	1.5	1960	115	0.6
C1-naphthalenes	133	103	2.0	1370	106	0.7
C2-naphthalenes	144	103	3.7	1360	97.6	1.7
C3-naphthalenes	191	99.1	2.5	1850	95.9	1.8
C4-naphthalenes	214	101	5.2	1990	94.4	2.1
acenaphthylene-d_8*	Internal Standard					
fluorene	23.1	92.4	1.1	246	98.8	1.1
C1-fluorenes	35.3	92.3	3.8	359	95.7	2.5
C2-fluorenes	38.9	82.8	7.8	457	96.8	5.1
C3-fluorenes^	304	89.9	3.3	310	91.7	5.0
phenanthrene-d_{10}	Internal Standard					
phenanthrene	79.2	99.0	1.7	836	105	1.6
C1-phenanthrenes & anthracenes	69.1	98.7	2.3	682	97.5	1.5
C2-phenanthrenes & anthracenes	109	107	3.7	979	96.5	3.5
C3-phenanthrenes & anthracenes^	897	90.6	1.5	879	88.7	2.6
C4-phenanthrenes & anthracenes^	832	116	11.4	498	74.4	8.7
phenanthrene-d_{10}	Internal Standard					
dibenzothiophene[1]	7.8	98.7	1.0	8.1	103	3.4
C1-dibenzothiophenes[1]	10.3	103	5.9	9.6	96.3	9.4
C2-dibenzothiophenes[1]	6.7	95.6	8.1	6.2	89.6	10.7
C3-dibenzothiophenes^	32.4	119	19.1	32.0	118	15.6
fluoranthene-d_{10}	Internal Standard					
fluoranthene	23.6	103	0.8	240	104	0.6
C1-fluoranthenes & pyrenes	40.2	89.3	6.2	437	97.0	1.2
C2-fluoranthenes & pyrenes^	Below Detection			4.7	93.6	15.0
chrysene-d_{12}	Internal Standard					
chrysene & triphenylene	8.7	97.2	3.2	89.7	103	2.9
C1-benz[a]anthracenes & chrysenes & triphenylenes	10.6	106	6.3	100	99.6	3.0
C2-benz[a]anthracenes & chrysenes & triphenylenes^	4.1	82.6	5.3	4.9	97.1	7.1

Relative standard deviations (%RSD) for the inter-day study were calculated by multiplying the division of the standard deviation by the average concentration of either a 1:200 dilution of SRM 1991; or a 1:20 dilution of SRM 1991 ran 8 times (indicated by ^) by 100. The average and %recovery for the intra-day study were calculated using a 1:20 dilution of SRM 1991; or a 1:200 dilution of SRM 1991 (indicated by [1]) ran 8 times across three days.

Table S5. Low Density Polyethylene Matrix Spike with SRM 1991

Analyte	SRM Mass (mg/kg)		SRM Concentration (pg/μL)	% Recovery	Uncertainty Range[a]
naphthalene	26	±1.1	3460	74.6	N/A
C1-naphthalenes	19.7	±35	2620	84.6	N/A
C2-naphthalenes	21.3	±2.8	2830	90.0	N/A
C3-naphthalenes	29.5	±1.5	3920	110	N/A
C4-naphthalenes	33.8	±4.7	4490	119	N/A
fluorene	N/A	N/A	N/A	N/A	N/A
C1-fluorenes	5.62	±0.27	747	84.4	N/A
C2-fluorenes	7.2	±0.39	958	91.9	N/A
C3-fluorenes	5.16	±0.50	686	108	N/A
phenanthrene	12.1	±27	1610	72.1	N/A
C1-phenanthrenes & anthracenes	10.7	±3.2	1420	71.0	N/A
C2-phenanthrenes & anthracenes	15.5	±1.9	2060	69.4	79.1% within range
C3-phenanthrenes & anthracenes	15.1	±0.2	2010	92.1	N/A
C4-phenanthrenes & anthracenes	10.2	±1.1	1360	not detected	N/A
dibenzothiophene	1.2	±0.06	160	64.5	outside range
C1-dibenzothiophenes	1.53	±0.04	203	64.4	outside range
C2-dibenzothiophenes	1.06	±0.09	141	79.4	N/A
C3-dibenzothiophenes	0.415	±0.036	55.2	not detected	N/A
fluoranthene	3.54	±0.39	471	69.7	78.3% within range
C1-fluoranthenes & pyrenes	6.86	±0.54	912	72.3	N/A
C2-fluoranthenes & pyrenes	1.67	±0.44	222	3.51	outside range
chrysene & triphenylene	1.32	±0.15	175	64.9	73.3% within range
C1-benz[a]anthracenes & chrysenes & triphenylenes	1.54	±0.46	205	54.7	78.0% within range
C2-benz[a]anthracenes & chrysenes & triphenylenes	0.44	±0.12	58.5	25.6	outside range

[a]*Error range: determined as the concentration range of SRM 1991 with the associated uncertainty in the certified mass values. Percent recoveries falling outside data quality objectives of ±30% of the expected values were recalculated taking into account the uncertainty range. If within the uncertainty, the analyte was considered within data quality object*

Table S6. SRM 2779 Percent Recovery

**Analyte**	**SRM Mass (mg/kg)**		**SRM Concentration (pg/µL)**	**% Recovery**
naphthalene	855	±46	89200	92.1
C1-naphthalenes	2770	±35	289000	83.5
C2-naphthalenes	2170	±360	304000[a]	139
C3-naphthalenes	1380	±270	85400[a]	*
C4-naphthalenes	700	±130	101000[a]	*
fluorene	145	±43	15100	80.2
C1-fluorenes	300	±60	44300[a]	149
C2-fluorenes	380	±30	46200[a]	*
C3-fluorenes	270	±40	36800[a]	*
phenanthrene	258	±27	26900	84.1
C1-phenanthrenes & anthracenes	670	±90	89400[a]	91.5
C2-phenanthrenes & anthracenes	630	±60	52700[a]	*
C3-phenanthrenes & anthracenes	400	±50	52600[a]	*
C4-phenanthrenes & anthracenes	200	±30	27400[a]	*
dibenzothiophene	51.8	±2.1	5400	104
C1-dibenzothiophenes	130	±20	17900[a]	105
C2-dibenzothiophenes	160	±20	21000[a]	103
C3-dibenzothiophenes	110	±10	9290[a]	78.1
fluoranthene	4.36	±0.4	455	234
C1-fluoranthenes & pyrenes	67.0	±7	8520[a]	142
C2-fluoranthenes & pyrenes	130	±20	9180[a]	9.63
chrysene & triphenylene	47.4	±1.7	4940	67.1
C1-benz[a]anthracenes & chrysenes & triphenylenes	110	±7	99500[a]	94.9
C2-benz[a]anthracenes & chrysenes & triphenylenes	130	±10	11400[a]	20.5

[a]Determined as the concentration of SRM 2779 with the associated expanded uncertainty in the reference mass values as listed on the NIST certificate of analysis.
**Interferences present, percent recovery not able to be calculated.*

Table S7. SRM 2779 Ratio Results

Abbreviation	Pyro	Petro	Ratio Value	Score for Pyrogenic	Score for Petrogenic
A0/PA0	> 0.1 (3)	< 0.1 (3)	N/A	N/A	N/A
P0/A0	< 5 (3)	> 30 (3)	N/A	N/A	N/A
PA1/PA0	< 1 ✓	> 1.5 (2)	N/A	N/A	N/A
PA0/PA01	> 0.5 (1)	≤ 0.4 (1)	N/A	N/A	N/A
FL0/PY0	> 1 (1)	≤ 0.5 (1)	0.00	N/A	1
FL0/FLPY	> 0.5 (1)	< 0.4 (2)	0.00	N/A	2
FLPY0/FLPY01	> 0.5 (3)	< 0.5 (3)	0.112	N/A	3
FLP1/PY0	~ 0.3 (1)	~ 4 (1)	7.97	N/A	N/A
FLP1/FLPY0	< 1 (2)	> 1 (1)	7.97	N/A	1
FLPY/(P2 + P3 + P4)	< 0.3 (1)	> 9 (1)	N/A	N/A	N/A
BaA/Ch0	≥ 0.5 (1)	< 0.25 (1)	0.00	N/A	1
BaA/228	> 0.35 (1)	< 0.2 (1)	0.00	N/A	1
D2/P2	< 0.4 (1)	N/A	0.071	1	N/A
D3/P3	< 0.4 (1)	N/A	0.013	1	N/A
PY0/BaP	< 10 (1)	≥ 10 (1)	1.49	1	N/A
IP/ghi	> 1 (1)	< 0.25 (2)	N/A	N/A	N/A
IP/IP + ghi	> 0.5 (1)	< 0.2 (1)	N/A	N/A	N/A
BeP/BaP	< 1 (1)	N/A	0.00	1	N/A
∑alkyl/PAHs	<1 (2)	> 2.3 (1)	36.1	N/A	1
L/H	< 1 (1)	> 1 (1)	10.1	N/A	1
LPAH/HPAH	< 0.4 (1)	> 2.3 (1)	14.6	N/A	1
pyrogenic index	0.8 ↔ 2 (2)	<0.05 (2)	0.023	N/A	2
Sum Score	N/A	N/A	N/A	4	**14**

Table S8. SRM 1582 Percent Recovery

Analyte	SRM Mass (mg/kg)		SRM Concentration (pg/µL)	% Recovery
naphthalene	153	±13	15300	92.6
C1-naphthalenes	620	±90	71300[a]	104
C2-naphthalenes	1100	±170	127000[a]	166
C3-naphthalenes	1100	±200	130000[a]	*
C4-naphthalenes	770	±150	92300[a]	*
fluorene	37	±11	3700	89.2
C1-fluorenes	120	±20	14200[a]	212
C2-fluorenes	230	±40	27200[a]	*
C3-fluorenes	230	±20	25200[a]	*
phenanthrene	99	±15	9910	94.9
C1-phenanthrenes & anthracenes	350	±50	40200[a]	123
C2-phenanthrenes & anthracenes	470	±50	52300[a]	*
C3-phenanthrenes & anthracenes	430	±50	48300[a]	*
C4-phenanthrenes & anthracenes	230	±40	27200[a]	*
dibenzothiophene	46	±15	4600	85.1
C1-dibenzothiophenes	120	±16	13800[a]	104
C2-dibenzothiophenes	190	±30	22200[a]	136
C3-dibenzothiophenes	170	±20	14800[a]	75.2
fluoranthene	3.62	±0.78	519[a]	180
C1-fluoranthenes & pyrenes	52	±7	6120[a]	210
C2-fluoranthenes & pyrenes	96	±10	8400[a]	8.10
chrysene & triphenylene	18.2	±1.4	1820[a]	73.7
C1-benz[a]anthracenes & chrysenes & triphenylenes	54	±5	6120[a]	128
C2-benz[a]anthracenes & chrysenes & triphenylenes	100	±15	8300[a]	122.6

[a]Determined as the concentration of SRM 1582 with the associated expanded uncertainty in the reference mass values as listed on the NIST certificate of analysis.
**Interferences present, percent recovery not able to be calculated.*

Table S9. SRM 1582 Ratio Results

Abbreviation	Pyro	Petro	Ratio Value	Score for Pyrogenic	Score for Petrogenic
A0/PA0	> 0.1 (3)	< 0.1 (3)	N/A	N/A	N/A
P0/A0	< 5 (3)	> 30 (3)	N/A	N/A	N/A
PA1/PA0	< 1 (1)	> 1.5 (2)	N/A	N/A	N/A
PA0/PA01	> 0.5 (1)	≤ 0.4 (1)	N/A	N/A	N/A
FL0/PY0	> 1 (1)	≤ 0.5 (1)	0.966	N/A	N/A
FL0/FLPY	> 0.5 (1)	< 0.4 (2)	0.491	N/A	N/A
FLPY0/FLPY01	> 0.5 (3)	< 0.5 (3)	0.108	N/A	3
FLP1/PY0	~ 0.3 (1)	~ 4 (1)	16.2	N/A	N/A
FLP1/FLPY0	< 1 (2)	> 1 (1)	8.24	N/A	1
FLPY/(P2 + P3 + P4)	< 0.3 (1)	> 9 (1)	0.00107	1	N/A
BaA/Ch0	≥ 0.5 (1)	< 0.25 (1)	0.359	N/A	N/A
BaA/228	> 0.35 (1)	< 0.2 (1)	0.189	N/A	1
D2/P2	< 0.4 (1)	N/A	0.110	1	N/A
D3/P3	< 0.4 (1)	N/A	0.017	1	N/A
PY0/BaP	< 10 (1)	≥ 10 (1)	N/A	N/A	N/A
IP/ghi	> 1 (1)	< 0.25 (2)	0.00	N/A	2
IP/IP + ghi	> 0.5 (1)	< 0.2 (1)	0.00	N/A	1
BeP/BaP	< 1 (1)	N/A	N/A	N/A	N/A
∑alkyl/PAHs	<1 (2)	> 2.3 (1)	112	N/A	1
L/H	< 1 (1)	> 1 (1)	7.16	N/A	1
LPAH/HPAH	< 0.4 (1)	> 2.3 (1)	8.60	N/A	1
pyrogenic index	0.8 ⟷ 2 (2)	<0.05 (2)	0.052	N/A	N/A
Sum Score	N/A	N/A	N/A	3	**11**

Table S10. SRM 1580 Percent Recovery

Analyte	SRM Mass (mg/kg)		SRM Concentration (pg/µL)	% Recovery	Additional Detections (pg/µL)
naphthalene	889	±46	93500	106	N/A
C1-naphthalenes	N/A	N/A	N/A	N/A	109000
C2-naphthalenes	N/A	N/A	N/A	N/A	2845000
C3-naphthalenes	N/A	N/A	N/A	N/A	901000
C4-naphthalenes	N/A	N/A	N/A	N/A	1570000
fluorene	N/A	N/A	N/A	N/A	10000
C1-fluorenes	N/A	N/A	N/A	N/A	40000
C2-fluorenes	N/A	N/A	N/A	N/A	270000
C3-fluorenes	N/A	N/A	N/A	N/A	214000
phenanthrene	274	±22	18700[a]	120	N/A
C1-phenanthrenes & anthracenes	N/A	N/A	N/A	N/A	54100
C2-phenanthrenes & anthracenes	N/A	N/A	N/A	N/A	255000
C3-phenanthrenes & anthracenes	N/A	N/A	N/A	N/A	775000
C4-phenanthrenes & anthracenes	N/A	N/A	N/A	N/A	564000
dibenzothiophene	N/A	N/A	N/A	N/A	2230
C1-dibenzothiophenes	N/A	N/A	N/A	N/A	6630
C2-dibenzothiophenes	N/A	N/A	N/A	N/A	11900
C3-dibenzothiophenes	N/A	N/A	N/A	N/A	6230
fluoranthene	53.1	±1.0	5580	106	N/A
C1-fluoranthenes & pyrenes	N/A	N/A	N/A	N/A	34600
C2-fluoranthenes & pyrenes	N/A	N/A	N/A	N/A	1270
chrysene & triphenylene	38.4	±9	4040	73.0	N/A
C1-benz[a]anthracenes & chrysenes & triphenylenes	N/A	N/A	N/A	N/A	17500
C2-benz[a]anthracenes & chrysenes & triphenylenes	N/A	N/A	N/A	N/A	2940

[a]*Determined as the concentration of SRM 1580 with the associated expanded uncertainty in the reference mass values as listed on the NIST certificate of analysis.*

Table S11. SRM 1580 Ratio Results

Abbreviation	Pyro	Petro	Ratio Value	Score for Pyrogenic	Score for Petrogenic
A0/PA0	> 0.1 (3)	< 0.1 (3)	0.217	3	N/A
P0/A0	< 5 (3)	> 30 (3)	3.61	3	N/A
PA1/PA0	< 1 (1)	> 1.5 (2)	1.93	N/A	2
PA0/PA01	> 0.5 (1)	≤ 0.4 (1)	0.341	N/A	1
FL0/PY0	> 1 (1)	≤ 0.5 (1)	0.662	N/A	N/A
FL0/FLPY	> 0.5 (1)	< 0.4 (2)	0.398	N/A	2
FLPY0/FLPY01	> 0.5 (3)	< 0.5 (3)	0.263	N/A	3
FLP1/PY0	~ 0.3 (1)	~ 4 (1)	4.66	N/A	1
FLP1/FLPY0	< 1 (2)	> 1 (1)	2.80	N/A	1
FLPY/(P2 + P3 + P4)	< 0.3 (1)	> 9 (1)	0.00733	1	N/A
BaA/Ch0	≥ 0.5 (1)	< 0.25 (1)	1.01	2	N/A
BaA/228	> 0.35 (1)	< 0.2 (1)	0.449	1	N/A
D2/P2	< 0.4 (1)	N/A	0.051	1	N/A
D3/P3	< 0.4 (1)	N/A	0.011	1	N/A
PY0/BaP	< 10 (1)	≥ 10 (1)	5.02	1	N/A
IP/ghi	> 1 (1)	< 0.25 (2)	0.728	N/A	N/A
IP/IP + ghi	> 0.5 (1)	< 0.2 (1)	0.421	N/A	N/A
BeP/BaP	< 1 (1)		1.06	N/A	N/A
∑alkyl/PAHs	<1 (2)	> 2.3 (1)	33.3	N/A	1
L/H	< 1 (1)	> 1 (1)	4.71	N/A	1
LPAH/HPAH	< 0.4 (1)	> 2.3 (1)	6.64	N/A	1
pyrogenic index	0.8 ⟷ 2 (2)	<0.05 (2)	0.113	N/A	N/A
Sum Score	N/A	N/A	N/A	13	13

Table S12. SRM 1597a Percent Recovery

Analyte	SRM Mass (mg/kg)		SRM Concentration (pg/µL)	% Recovery	Additional Detections (pg/µL)
naphthalene	1030	±100	896000	118	N/A
C1-naphthalenes	138.9	±2.4	123000[a]	104	N/A
C2-naphthalenes	5.75	±0.63	5550[a]	*	N/A
C3-naphthalenes	N/A	N/A	N/A	N/A	30000
C4-naphthalenes	N/A	N/A	N/A	N/A	19200
fluorene	145	±4	126000[a]	109	N/A
C1-fluorenes	N/A	N/A	N/A	N/A	30900
C2-fluorenes	N/A	N/A	N/A	N/A	12400
C3-fluorenes	N/A	N/A	N/A	N/A	N/A
phenanthrene	454	±7	395000	115	N/A
C1-phenanthrenes & anthracenes	60.9	±0.5	53400[a]	123	N/A
C2-phenanthrenes & anthracenes	6.25	±0.20	5610[a]	*	N/A
C3-phenanthrenes & anthracenes	N/A	N/A	N/A	N/A	31300
C4-phenanthrenes & anthracenes	N/A	N/A	N/A	N/A	N/A
dibenzothiophene	17.7	±0.4	15700[a]	141	N/A
C1-dibenzothiophenes	4.16	±0.17	3770[a]	120	N/A
C2-dibenzothiophenes	N/A	N/A	N/A	N/A	1380
C3-dibenzothiophenes	N/A	N/A	N/A	N/A	N/A
fluoranthene	327	±0.7	284000	107	N/A
C1-fluoranthenes & pyrenes	25.1	±1.1	22800[a]	*	N/A
C2-fluoranthenes & pyrenes	N/A	N/A	N/A	N/A	709
chrysene & triphenylene	78.3	±2.9	68100	102	N/A
C1-benz[a]anthracenes & chrysenes & triphenylenes	2.57	±0.03	2260[a]	*	N/A
C2-benz[a]anthracenes & chrysenes & triphenylenes	N/A	N/A	N/A	N/A	530

[a]*Determined as the concentration of SRM 1597a with the associated expanded uncertainty in the reference mass values as listed on the NIST certificate of analysis.*
**Interferences present, percent recovery not able to be calculated*

Table S13. SRM 1597a Ratio Results

Abbreviation	Pyro	Petro	Ratio Value	Score for Pyrogenic	Score for Petrogenic
A0/PA0	> 0.1 (3)	< 0.1 (3)	0.183	3	N/A
P0/A0	< 5 (3)	> 30 (3)	4.48	3	N/A
PA1/PA0	< 1 (1)	> 1.5 (2)	0.0820	1	N/A
PA0/PA01	> 0.5 (1)	≤ 0.4 (1)	0.925	1	N/A
FL0/PY0	> 1 (1)	≤ 0.5 (1)	1.36	1	N/A
FL0/FLPY	> 0.5 (1)	< 0.4 (2)	0.575	1	N/A
FLPY0/FLPY01	> 0.5 (3)	< 0.5 (3)	0.855	3	N/A
FLP1/PY0	~ 0.3 (1)	~ 4 (1)	0.400	N/A	N/A
FLP1/FLPY0	< 1 (2)	> 1 (1)	0.170	3	N/A
FLPY/(P2 + P3 + P4)	< 0.3 (1)	> 9 (1)	N/A	N/A	N/A
BaA/Ch0	≥ 0.5 (1)	< 0.25 (1)	1.63	2	N/A
BaA/228	> 0.35 (1)	< 0.2 (1)	0.532	1	N/A
D2/P2	< 0.4 (1)	N/A	0.031	1	N/A
D3/P3	< 0.4 (1)	N/A	N/A	N/A	N/A
PY0/BaP	< 10 (1)	≥ 10 (1)	10.6	N/A	1
IP/ghi	> 1 (1)	< 0.25 (2)	0.747	N/A	N/A
IP/IP + ghi	> 0.5 (1)	< 0.2 (1)	0.427	N/A	N/A
BeP/BaP	< 1 (1)	N/A	2.39	N/A	N/A
∑alkyl/PAHs	<1 (2)	> 2.3 (1)	0.201	2	N/A
L/H	< 1 (1)	> 1 (1)	3.59	N/A	1
LPAH/HPAH	< 0.4 (1)	> 2.3 (1)	1.81	N/A	N/A
pyrogenic index	0.8 ⟷ 2 (2)	<0.05 (2)	7.27	N/A	N/A
Sum Score	N/A	N/A	N/A	**22**	2

Table S14. SRM 1975 Percent Recovery

Analyte	SRM Mass (mg/kg)		SRM Concentration (pg/μL)	% Recovery	Additional Detections (pg/μL)
naphthalene	0.67	±0.01	878[a]	74.8	N/A
C1-naphthalenes	1.08	±0.02	1410[a]	63.3	N/A
C2-naphthalenes	N/A	N/A	N/A	N/A	2100
C3-naphthalenes	N/A	N/A	N/A	N/A	3540
C4-naphthalenes	N/A	N/A	N/A	N/A	2250
fluorene	0.11	±0.003	142[a]	62.0	N/A
C1-fluorenes	N/A	N/A	N/A	N/A	161
C2-fluorenes	N/A	N/A	N/A	N/A	264
C3-fluorenes	N/A	N/A	N/A	N/A	2230
phenanthrene	8	±0.2	10600	90.0	N/A
C1-phenanthrenes & anthracenes	3.33	±0.06	4350[a]	75.7	N/A
C2-phenanthrenes & anthracenes	0.662	±0.007	890[a]	*	N/A
C3-phenanthrenes & anthracenes	N/A	N/A	N/A	N/A	2760
C4-phenanthrenes & anthracenes	N/A	N/A	N/A	N/A	N/A
dibenzothiophene	N/A	N/A	N/A	N/A	600
C1-dibenzothiophenes	N/A	N/A	N/A	N/A	1090
C2-dibenzothiophenes	N/A	N/A	N/A	N/A	1250
C3-dibenzothiophenes	N/A	N/A	N/A	N/A	391
fluoranthene	13.5	±0.6	17900	105	N/A
C1-fluoranthenes & pyrenes	0.092	±0.004	128[a]	*	N/A
C2-fluoranthenes & pyrenes	N/A	N/A	N/A	N/A	N/A
chrysene & triphenylene	4.33	±0.09	5760	83.0	N/A
C1-benz[a]anthracenes & chrysenes & triphenylenes	N/A	N/A	N/A	N/A	N/A
C2-benz[a]anthracenes & chrysenes & triphenylenes	N/A	N/A	N/A	N/A	N/A

[a]Determined as the concentration of SRM 1975 with the associated expanded uncertainty in the reference mass values as listed on the NIST certificate of analysis.
**Interferences present, percent recovery not able to be calculated.*

Table S15. SRM 1975 Ratio Results

Abbreviation	Pyro	Petro	Ratio Value	Score for Pyrogenic	Score for Petrogenic
A0/PA0	> 0.1 (3)	< 0.1 (3)	N/A	N/A	N/A
P0/A0	< 5 (3)	> 30 (3)	N/A	N/A	N/A
PA1/PA0	< 1 (1)	> 1.5 (2)	N/A	N/A	N/A
PA0/PA01	> 0.5 (1)	≤ 0.4 (1)	N/A	N/A	N/A
FL0/PY0	> 1 (1)	≤ 0.5 (1)	34.0	1	N/A
FL0/FLPY	> 0.5 (1)	< 0.4 (2)	0.971	1	N/A
FLPY0/FLPY01	> 0.5 (3)	< 0.5 (3)	0.973	3	N/A
FLP1/PY0	~ 0.3 (1)	~ 4 (1)	0.964	N/A	N/A
FLP1/FLPY0	< 1 (2)	> 1 (1)	0.028	3	N/A
FLPY/(P2 + P3 + P4)	< 0.3 (1)	> 9 (1)	N/A	N/A	N/A
BaA/Ch0	≥ 0.5 (1)	< 0.25 (1)	0.041	N/A	1
BaA/228	> 0.35 (1)	< 0.2 (1)	0.019	N/A	1
D2/P2	< 0.4 (1)	N/A	0.290	1	N/A
D3/P3	< 0.4 (1)	N/A	0.142	1	N/A
PY0/BaP	< 10 (1)	≥ 10 (1)	N/A	N/A	N/A
IP/ghi	> 1 (1)	< 0.25 (2)	N/A	N/A	N/A
IP/IP + ghi	> 0.5 (1)	< 0.2 (1)	N/A	N/A	N/A
BeP/BaP	< 1 (1)	N/A	N/A	N/A	N/A
∑alkyl/PAHs	<1 (2)	> 2.3 (1)	0.595	2	N/A
L/H	< 1 (1)	> 1 (1)	9.10	N/A	1
LPAH/HPAH	< 0.4 (1)	> 2.3 (1)	0.452	N/A	N/A
pyrogenic index	0.8 ⟷ 2 (2)	<0.05 (2)	4.51	N/A	N/A
Sum Score	N/A	N/A	N/A	**12**	2

Table S16. SRM Instrument Concentrations

	Instrument Concentration in pg/µL				
**Analyte**	**SRM 2779**	**SRM 1580**	**SRM 1582**	**SRM 1597a**	**SRM 1975**
naphthalene	82100	129000	15100	1060000	657
C1-naphthalenes	241000	145000	74400	127000	892
C2-naphthalenes	424000	341000	211000	44000	2100
C3-naphthalenes	908000	1100000	701000	29900	3540
C4-naphthalenes	982000	1740000	1270000	19200	2250
fluorene	12100	12600	3301	137000	88.2
C1-fluorenes	65800	52700	30100	30900	161
C2-fluorenes	248000	347000	193000	12400	264
C3-fluorenes	145000	304000	134000	<44.5	2230
phenanthrene	22600	30400	9400	453000	9570
C1-phenanthrenes & anthracenes	81800	64600	49300	65700	3290
C2-phenanthrenes & anthracenes	308000	285000	275000	44400	4320
C3-phenanthrenes & anthracenes	543000	818000	662000	31300	2760
C4-phenanthrenes & anthracenes	422000	665000	523000	<125	<125
dibenzothiophene	5640	4330	3920	22200	601
C1-dibenzothiophenes	14300	9360	14400	4520	1090
C2-dibenzothiophenes	21700	14400	30200	1380	1250
C3-dibenzothiophenes	7260	9130	11100	<14.6	391
fluoranthene	1070	7100	934	305000	9790
C1-fluoranthenes & pyrenes	12100	36400	12800	128000	488
C2-fluoranthenes & pyrenes	884	1140	682	708	<2.04
chrysene & triphenylene	3310	4100	1340	69100	4780
C1-benz[a]anthracenes & chrysenes & triphenylenes	10900	24600	7810	17400	<0.67
C2-benz[a]anthracenes & chrysenes & triphenylenes	2340	3070	1880	530	<1.00
naphthalene*	69400	92100	15400	1060000	799
acenaphthylene*	<2.33	5120	<2.33	314000	166
acenaphthene*	<1.07	19400	8400	12200	<1.07
fluorene*	12000	12900	3680	189000	135
dibenzothiophene*	3900	2490	3460	24200	766
phenanthrene*	26100	26200	11200	658000	11700
anthracene*	<1.05	7250	<1.05	147000	<1.05

fluoranthene*	<0.54	5160	764	432000	17200
pyrene*	1520	7800	792	319000	507
benz[a]anthracene*	<0.75	3150	426	147000	126
chrysene*	1720	3120	1180	90100	3110
benzo(b)fluoranthene*	770	982	530	88200	3880
benzo(k)fluoranthene*	<0.53	516	<0.53	56000	<0.53
benzo(e)pyrene*	<0.71	1640	430	71800	328
benzo(a)pyrene*	1020	1550	<1.18	30100	<1.18
perylene*	<1.00	<1.00	2460	28500	<1.00
indeno(1,2,3-c,d)pyrene*	<0.26	500	<0.26	42600	<0.26
dibenz(a,h)anthracene*	<1.02	323	<1.02	11200	<1.02
benzo(ghi)perylene*	<0.34	687	173	57100	<0.34
triphenylene*	2300	753	637	39300	3540

*Compounds indicated by a * are obtained from the 63 PAH method[2]*

Table S17. Deepwater Horizon Environmental Water Concentrations

	Water Concentration in ng/L					
**Analyte**	**Gulfport, MS June**	**Gulfport, MS June**	**Gulfport, MS August**	**Pensacola, FL August**	**Grand Isle, LA June**	**Grand Isle, LA June**
naphthalene	26.8	1.49	0.0605	0.0850	36.6	9.02
C1-naphthalenes	2.86	54.5	3.90	3.31	40.2	83.7
C2-naphthalenes	16.0	412	12.6	18.0	320	2410
C3-naphthalenes	222	1630	88.7	111	18000	42100
C4-naphthalenes	632	1757	308	270	91300	95700
fluorene	0.0548	0.0926	0.0150	0.0161	67.6	354
C1-fluorenes	1.77	12.5	0.712	1.56	3150	8850
C2-fluorenes	11.9	44.0	4.52	9.45	48200	59700
C3-fluorenes	11.7	25.4	27.1	0.481	71000	41700
phenanthrene	0.774	2.12	0.574	1.63	37.0	244
C1-phenanthrenes & anthracenes	5.31	6.61	12.4	7.89	1090	2320
C2-phenanthrenes & anthracenes	11.1	16.3	7.09	9.43	12800	10500
C3-phenanthrenes & anthracenes	15.6	13.3	12.4	9.04	23400	12600
C4-phenanthrenes & anthracenes	15.0	6.89	15.3	0.481	15700	7230
dibenzothiophene	0.0915	0.544	0.0585	0.0275	8.68	45.0

C1-dibenzothiophenes	0.398	2.07	0.192	0.190	187	424
C2-dibenzothiophenes	0.280	2.37	0.656	0.418	999	795
C3-dibenzothiophenes	0.415	0.584	0.489	0.215	476	231
fluoranthene	0.417	0.126	0.391	0.201	4.23	5.32
C1-fluoranthenes & pyrenes	0.498	0.152	0.340	0.293	40.8	35.4
C2-fluoranthenes & pyrenes	0.00473	0.00118	0.00314	0.00331	1.47	0.982
chrysene & triphenylene	0.0828	0.0216	0.0884	0.127	19.4	27.0
C1-benz[a]anthracenes & chrysenes & triphenylenes	0.0488	0.0105	0.0463	0.0540	27.9	34.4
C2-benz[a]anthracenes & chrysenes & triphenylenes	0.00436	0.000838	0.00497	0.00573	2.40	3.46
naphthalene*	30.9	2.75	1.50	1.35	4.66	0.565
acenaphthylene*	0.227	0.0216	0.356	0.0208	0.210	0.230
acenaphthene*	1.68	0.0105	1.03	0.0101	0.102	0.102
fluorene*	0.727	2.27	0.316	0.340	0.0362	4.61
dibenzothiophene*	0.0700	0.474	0.0414	0.0132	0.494	3.90
phenanthrene*	0.914	2.37	0.638	0.516	4.24	27.6
anthracene*	0.327	2.92	0.200	0.119	0.0226	0.0226
fluoranthene*	1.14	0.424	0.883	0.599	0.689	0.936
pyrene*	1.21	0.329	1.06	0.451	1.80	1.36
benz[a]anthracene*	0.0537	0.0137	0.0430	0.0311	0.000919	0.00217
chrysene*	0.0408	0.00814	0.0621	0.0453	0.675	1.00
benzo(b)fluoranthene*	0.0352	0.00514	0.0421	0.0373	0.113	0.143
benzo(k)fluoranthene*	0.0133	0.00205	1.26	2.09	0.00061	0.00161
benzo(e)pyrene*	0.0301	0.00260	2.83	4.54	0.00935	0.275
benzo(a)pyrene*	0.0120	0.00101	0.192	0.387	0.00136	0.00361
perylene*	0.0277	0.00297	0.0933	0.000469	0.00539	0.0743
indeno(1,2,3-c,d)pyrene*	0.00758	0.000107	0.0107	0.0133	0.000371	0.00103
dibenz(a,h)anthracene*	0.00227	0.00043	0.00272	0.000618	0.00150	0.00418
benzo(ghi)perylene*	0.00714	0.00204	0.0106	0.0115	0.00793	0.0227
triphenylene*	0.0736	0.0157	0.0732	0.112	2.15	2.33

*Compounds indicated by a * are obtained from the 63 PAH method[2]*

Table S18. St Helens Air Concentrations

	Air Concentration in ng/m^3				
**Analyte**	**Site 3**	**Site 9 Rep1**	**Site 9 Rep2**	**Site 9 Rep3**	**Site 12**
naphthalene	92.1	291	350	333	331
C1-naphthalenes	115	660	745	863	996
C2-naphthalenes	492	1400	1480	1710	4490
C3-naphthalenes	2550	4650	4760	5380	18000
C4-naphthalenes	10900	16800	17100	19100	63200
fluorene	8.93	24.1	24.7	26.7	91.3
C1-fluorenes	13.5	22.8	29.3	25.4	118
C2-fluorenes	35.4	47.5	50.7	51.3	221
C3-fluorenes	15.8	24.6	23.2	21.5	73.9
phenanthrene	5.34	9.93	6.89	6.89	63.8
C1-phenanthrenes & anthracenes	1.66	2.84	10.1	11.0	19.9
C2-phenanthrenes & anthracenes	2.54	3.94	2.89	3.05	21.2
C3-phenanthrenes & anthracenes	2.43	3.05	3.88	4.07	11.9
C4-phenanthrenes & anthracenes	<0.225	<0.225	2.81	2.74	<0.225
dibenzothiophene	0.172	0.329	0.330	0.365	2.06
C1-dibenzothiophenes	0.102	0.188	0.180	0.212	1.30
C2-dibenzothiophenes	0.0776	0.112	0.119	0.127	0.521
C3-dibenzothiophenes	0.0849	0.0858	0.0848	0.0866	0.0928
fluoranthene	0.0160	0.0544	0.0274	0.0666	0.538
C1-fluoranthenes & pyrenes	0.0107	0.0377	0.0192	0.0424	0.345
C2-fluoranthenes & pyrenes	0.000265	0.000403	0.000221	0.000459	<0.000700
chrysene & triphenylene	0.000970	0.00233	0.00106	0.00320	0.0173
C1-benz[a]anthracenes & chrysenes & triphenylenes	0.000823	0.00146	0.000915	0.00130	0.00502
C2-benz[a]anthracenes & chrysenes & triphenylenes	8.05E-05	0.000131	6.48E-05	0.000151	<0.000238
naphthalene*	60.0	188	235	242	205
acenaphthylene*	<0.0300	<0.0300	<0.0300	<0.0300	<0.0300
acenaphthene*	15.7	64.5	68.2	73.4	226
fluorene*	6.35	17.0	18.2	19.7	64.8
dibenzothiophene*	0.117	0.224	0.220	0.246	1.37
phenanthrene*	4.00	7.42	7.54	8.18	44.1
anthracene*	<0.00263	<0.00263	<0.00263	<0.00263	2.94
fluoranthene*	0.0666	0.198	0.105	0.243	2.04
pyrene*	0.0274	0.0873	0.0484	0.106	0.826
benz[a]anthracene*	0.000388	0.00154	0.000730	0.00203	0.0136
chrysene*	0.000566	0.00153	0.000664	0.00213	0.0115
benzo(b)fluoranthene*	0.000213	0.00037	0.000156	0.000639	0.00141
benzo(k)fluoranthene*	<3.57E-05	<3.57E-05	<3.57E-05	<3.57E-05	0.000475
benzo(e)pyrene*	9.55E-05	0.000171	5.91E-05	0.000281	0.000507
benzo(a)pyrene*	<9.50E-05	<9.50E-05	<9.50E-05	<9.50E-05	<9.50E-05
indeno(1,2,3-c,d)pyrene*	<1.64E-05	<1.64E-05	<1.64E-05	<1.64E-05	<1.64E-05
dibenz(a,h)anthracene*	<5.91E-05	<5.91E-05	<5.91E-05	<5.91E-05	<5.91E-05
benzo(ghi)perylene*	9.28E-05	0.000115	4.93E-05	0.000186	0.00031
triphenylene*	0.000287	0.000529	0.000244	0.000706	0.00366

*Compounds indicated by a * are obtained from the 63 PAH method[2]*

Table S19. St Helens Environmental Water Concentrations

Analyte	Water Concentration in ng/L									
	**Site 2**	**Site 3**	**Site 4**	**Site 9**	**Site 10 Rep1**	**Site 10 Rep2**	**Site 10 Rep3**	**Site 12**	**Site 13**	**Site 14**
naphthalene	12.6	2.84	3.62	5.74	13.5	38.4	11.0	7.23	11.2	5.36
C1-naphthalenes	39.6	7.58	10.2	19.4	31.8	82.2	26.4	20.1	27.7	9.3
C2-naphthalenes	132	43	61.8	105	120	313	106	85.4	96.8	44
C3-naphthalenes	460	454	515	832	815	2400	712	586	672	380
C4-naphthalenes	996	1940	2620	3250	3330	10400	3230	3240	3030	1430
fluorene	5.35	1.44	1.81	6.70	7.87	20.6	6.56	5.20	6.46	4.49
C1-fluorenes	10.9	7.25	8.68	19.8	17.8	53.5	15.8	11.4	15.5	10.0
C2-fluorenes	35.9	47.0	70.7	112	99.8	298	91.6	88.7	92.0	46.7
C3-fluorenes	32.1	47.3	80.0	102	94.5	282	90.6	96.0	91.6	42.8
phenanthrene	6.67	1.96	2.25	7.20	7.66	20.7	5.83	5.96	6.71	2.7
C1-phenanthrenes & anthracenes	5.99	4.83	5.62	12.5	12.0	36.6	10.2	11.1	12.6	4.56
C2-phenanthrenes & anthracenes	13.5	20.7	29.4	46.6	45.2	135	38.8	45.5	53.0	17.5
C3-phenanthrenes & anthracenes	19.1	30.9	42.4	62.5	57.6	176	50.1	61.4	82.5	25.2
C4-phenanthrenes & anthracenes	<0.276	9.51	9.24	24.4	17.3	56.5	14.6	23.6	39.3	9.87
dibenzothiophene	0.481	0.218	0.213	0.840	0.857	2.54	0.694	0.580	0.705	0.356
C1-dibenzothiophenes	0.129	0.627	0.666	1.77	1.61	5.22	1.05	1.11	1.54	0.550
C2-dibenzothiophenes	0.677	1.12	1.47	2.74	2.51	7.90	2.15	2.37	3.01	0.696
C3-dibenzothiophenes	0.282	0.448	0.531	1.22	0.694	3.32	0.677	0.688	0.901	0.238
fluoranthene	4.02	7.30	3.84	7.15	6.73	20.8	5.94	7.16	10.6	2.95
C1-fluoranthenes & pyrenes	1.46	3.45	2.08	3.82	3.57	11.1	3.15	4.47	6.89	1.19
C2-fluoranthenes & pyrenes	0.0237	0.0277	0.0114	0.0743	0.0673	0.219	0.0199	0.0260	0.0915	0.00932
chrysene & triphenylene	0.241	0.857	0.327	0.503	0.446	1.32	0.384	0.532	0.883	0.152
C1-benz[a]anthracenes & chrysenes & triphenylenes	0.152	0.429	0.144	0.219	0.192	0.586	0.158	0.222	0.372	0.0734
C2-benz[a]anthracenes & chrysenes & triphenylenes	0.00542	0.0239	0.00802	0.0100	0.00827	0.0255	0.00831	0.0104	0.0155	0.00412
naphthalene*	11.5	2.23	4.23	5.09	11.6	30.7	9.42	6.72	10.2	5.58
acenaphthylene*	0.401	0.298	0.292	0.849	0.833	1.89	0.883	0.936	1.38	0.468
acenaphthene*	9.52	2.67	3.51	15.9	22.0	58.4	17.3	12.8	15.7	9.30
fluorene*	4.12	1.96	2.20	6.83	8.05	22.0	6.83	5.52	6.69	3.88
dibenzothiophene*	0.282	0.118	0.120	0.589	0.605	1.79	0.475	0.503	0.564	0.240
phenanthrene*	5.50	1.35	1.69	6.27	6.71	18.5	5.56	5.63	5.76	2.41
anthracene*	0.581	0.520	0.558	2.02	1.74	5.27	1.44	1.72	2.68	0.870

fluoranthene*	3.63	2.50	3.35	6.94	6.42	20.6	5.53	6.65	10.2	2.59
pyrene*	4.51	3.47	4.94	8.71	8.55	27.0	7.58	9.22	13.4	3.61
benz[a]anthracene*	0.162	0.118	0.180	0.307	0.292	0.879	0.225	0.352	0.619	0.0801
chrysene*	0.168	0.137	0.207	0.315	0.287	0.922	0.247	0.328	0.565	0.0956
benzo(b)fluoranthene*	0.0447	0.0493	0.0651	0.111	0.0928	0.270	0.0729	0.118	0.208	0.0337
benzo(k)fluoranthene*	0.0200	0.0191	0.0276	0.0363	0.0394	0.111	0.0298	0.0491	0.091	0.0129
benzo(e)pyrene*	0.0407	0.0608	0.0802	0.119	0.0965	0.276	0.0765	0.116	0.204	0.0370
benzo(a)pyrene*	0.0161	0.0226	0.0298	0.0549	0.0477	0.153	0.0408	0.0664	0.123	0.0192
perylene*	0.0425	0.129	0.150	0.139	0.104	0.304	0.102	0.0969	0.126	0.0634
indeno(1,2,3-c,d)pyrene*	<8.52E-05	<8.52E-05	<8.52E-05	0.0130	0.0104	0.0256	0.00881	0.0116	0.0170	0.00472
dibenz(a,h)anthracene*	<0.000287	<0.000287	<0.000287	0.00347	0.00277	0.00726	0.00254	<0.000287	<0.000287	<0.000287
benzo(ghi)perylene*	0.00534	0.0106	0.0123	0.0166	0.0135	0.0370	0.0111	0.0156	0.0232	0.00738
triphenylene*	0.0911	0.0974	0.135	0.216	0.191	0.538	0.155	0.237	0.383	0.0708

*Compounds indicated by a * are obtained from the 63 PAH method[2]*

Table S20. St Helens Environmental Shallow Porewater Concentrations

	Porewater Concentration in ng/L														
**Analyte**	**Site 1**	**Site 2**	**Site 3**	**Site 4**	**Site 5**	**Site 6**	**Site 7**	**Site 8**	**Site 9**	**Site 10**	**Site 11**	**Site 12**	**Site 13**	**Site 14**	**Site 15**
naphthalene	2.40	84.8	2.40	2.68	926	3.45	5.76	10.5	5.41	5.47	6.14	669	2.40	5.36	3.60
C1-naphthalenes	5.01	1680	<2.45	10.2	24.7	9.83	8.6	16.1	4.22	5.24	6.44	366	2.58	10.9	6.62
C2-naphthalenes	21.8	24500	<4.85	20.7	99.6	18.6	16.9	35.3	18.9	28	18.9	449	18	32.4	20.7
C3-naphthalenes	94.3	44700	10.7	44.9	374	42	50.1	106	50.7	137	85.8	596	103	79.1	72.1
C4-naphthalenes	234	43500	73.1	150	541	93.3	104	239	118	409	234	795	388	170	180
fluorene	6.55	82.9	1.00	2.97	19.5	2.30	2.40	5.19	3.66	7.17	5.51	104	15.2	4.49	5.93
C1-fluorenes	4.84	206	0.341	0.691	3.46	0.99	1.34	3.53	1.7	5.04	3.21	37	9.58	3.01	3.26
C2-fluorenes	9.09	575	0.966	3.16	10.9	2.69	3.9	9.53	3.24	10.8	7.58	32.6	23.5	6.17	8.45
C3-fluorenes	6.94	306	<1.1	5.73	9.43	3.92	7.16	12.3	6.32	6.69	6.32	17.2	16.1	7.82	6.22
phenanthrene	1.55	95.4	0.521	3.25	3.54	0.817	0.854	1.80	1.03	1.18	1.64	85.9	1.53	2.78	12.3
C1-phenanthrenes & anthracenes	1.32	69.2	1.1	2.26	1.62	0.6	0.78	1.58	0.931	1.27	1.59	15.2	2.28	1.57	1.07
C2-phenanthrenes & anthracenes	3.27	148	1.81	2.8	4.47	1.77	1.93	4.79	2.66	3.93	4.44	15.1	7.64	3.9	3.57
C3-phenanthrenes & anthracenes	4.74	146	<0.746	3.35	6.28	3.08	<0.746	6.52	4.33	4.57	5.7	11.3	9.6	4.32	5.32
C4-phenanthrenes & anthracenes	1.44	63.7	<1.40	<1.40	2.56	<1.40	<1.40	2.41	<1.40	<1.40	<1.40	4.35	4.52	<1.40	<1.40
dibenzothiophene	0.708	20.4	0.362	0.600	0.350	0.234	0.248	0.537	0.384	0.732	0.616	7.95	1.03	0.356	0.396

C1-dibenzothiophenes	<0.0691	15.9	0.535	0.577	0.339	0.173	0.217	0.377	0.242	0.38	0.34	2.25	0.466	0.639	0.222
C2-dibenzothiophenes	0.328	10.8	0.319	0.325	0.342	0.21	0.232	0.335	0.283	0.312	0.369	0.932	0.507	0.492	0.302
C3-dibenzothiophenes	<0.934	2.86	<0.934	<0.934	<0.934	<0.934	<0.934	<0.934	<0.934	<0.934	<0.934	<0.934	<0.934	<0.934	<0.934
fluoranthene	0.830	5.16	0.184	1.67	0.880	0.419	0.375	0.874	0.676	1.02	1.31	12.7	2.87	2.95	1.05
C1-fluoranthenes & pyrenes	0.29	1.28	0.0886	0.11	0.208	0.197	0.164	0.395	0.284	0.341	0.545	2.69	1.12	0.302	0.394
C2-fluoranthenes & pyrenes	0.005	0.0419	<0.00441	<0.00441	<0.00441	<0.00441	<0.00441	0.00492	<0.00441	<0.00441	0.0042	0.00974	0.0112	<0.00441	<0.00441
chrysene & triphenylene	0.0368	1.25	0.0113	0.125	0.0905	0.0339	0.0207	0.0490	0.0445	0.0392	0.0843	0.566	0.166	0.152	0.0811
C1- benz[a]anthracenes & chrysenes & triphenylenes	0.0246	0.158	0.0129	0.00786	<0.00287	0.02	0.0129	0.0253	0.026	0.0237	0.0429	0.12	0.0541	0.0305	0.0782
C2-benz[a]anthracenes & chrysenes & triphenylenes	<0.00138	0.0162	<0.00138	<0.00138	<0.00138	<0.00138	<0.00138	<0.00138	<0.00138	<0.00138	<0.00138	0.00672	0.00426	<0.00138	<0.00138
naphthalene*	3.2	99	0.567	4.81	39	4.6	6.88	10.4	6.59	6.59	7.35	510	3.43	15.8	4.75
acenaphthylene*	<0.113	<0.113	<0.113	<0.113	<0.113	<0.113	<0.113	0.448	<0.113	<0.113	0.383	1.59	0.61	<0.113	<0.113
acenaphthene*	13	82.5	1.8	6.2	8.71	5.69	6.2	12.2	11.2	18.7	15.2	63	20.8	15.8	10.4
fluorene*	6.52	61.4	1.26	3.41	4.2	2.71	2.54	5.23	3.88	7.33	6.22	94	10.9	6.05	4.39
dibenzothiophene*	0.422	5.52	0.235	0.272	0.248	0.169	0.17	0.366	0.266	0.443	0.422	5.69	0.73	0.522	0.277
phenanthrene*	1.18	27.3	0.438	1.62	1.72	0.643	0.762	1.6	0.943	1.03	1.44	80.3	1.37	1.98	1.17
anthracene*	0.48	3.65	<0.0121	0.281	0.577	0.264	0.283	0.787	0.465	0.576	1.08	3.57	1.76	0.664	0.453
fluoranthene*	0.655	0.345	0.141	0.221	0.367	0.332	0.317	0.752	0.573	0.852	1.09	11.8	2.42	0.745	0.879
pyrene*	0.625	1.21	0.174	0.299	0.407	0.313	0.335	0.795	0.499	0.86	1.07	8.24	2.38	0.699	0.747
benz[a]anthracene*	0.0231	0.0308	<0.000932	0.00703	0.0161	0.0193	0.0124	0.0282	0.028	0.0242	0.0551	0.224	0.104	0.0296	0.0498
chrysene*	0.0247	0.0453	<0.000619	0.00694	0.0269	0.0194	0.011	0.0302	0.0314	0.025	0.0558	0.433	0.106	0.0288	0.0495
benzo(b)fluoranthene*	0.0104	0.00706	<0.000459	0.00356	0.0122	0.0114	0.00579	0.0118	0.0126	0.00887	0.0224	0.107	0.0372	0.0128	0.0237
benzo(k)fluoranthene*	<0.000710	<0.000710	<0.000710	<0.000710	<0.000710	<0.000710	0.00467	0.00709	0.00977	0.0067	0.013	0.058	0.0183	0.00952	0.0168
benzo(e)pyrene*	<0.00093	0.0101	<0.00093	<0.00093	0.0152	0.0136	0.00564	0.0121	0.0137	0.00895	0.022	0.098	0.0373	0.0146	0.024
benzo(a)pyrene*	<0.00160	<0.00160	<0.00160	<0.00160	<0.00160	<0.00160	<0.00160	0.00626	<0.00160	0.00621	0.0131	0.0516	0.0239	0.0105	0.0173
perylene*	0.0177	<0.00109	0.0187	<0.00109	0.0296	0.0239	0.0089	0.0126	0.0231	<0.00109	0.0146	0.0289	0.0166	0.0193	0.0579
indeno(1,2,3-c,d)pyrene*	<0.000490	<0.000490	<0.000490	<0.000490	<0.000490	<0.000490	<0.000490	<0.000490	<0.000490	<0.000490	<0.000490	<0.000490	<0.000490	<0.000490	<0.000490
dibenz(a,h)anthracene*	<0.0743	<0.0743	<0.0743	<0.0743	<0.0743	<0.0743	<0.0743	<0.0743	<0.0743	<0.0743	<0.0743	<0.0743	<0.0743	<0.0743	<0.0743
benzo(ghi)perylene*	<0.000560	<0.000560	<0.000560	<0.000560	<0.000560	<0.000560	<0.000560	<0.000560	<0.000560	<0.000560	<0.000560	0.0156	0.00882	<0.000560	0.0125
triphenylene*	0.0111	0.0158	<0.000563	0.00508	0.0221	0.0154	0.0108	0.0208	0.0178	0.0147	0.03	0.135	0.064	0.0176	0.0318

*Compounds indicated by a * are obtained from the 63 PAH method[2]*

Table S21. St Helens Environmental Deep Porewater Concentrations

	Porewater Concentration in ng/L														
**Analyte**	**Site 1**	**Site 2**	**Site 3**	**Site 4**	**Site 5**	**Site 6**	**Site 7**	**Site 8**	**Site 9**	**Site 10**	**Site 11**	**Site 12**	**Site 13**	**Site 14**	**Site 15**
naphthalene	<2.34	13.1	<2.34	2.34	2.339	2.34	2.34	2.34	2.34	2.34	2.36	8.12	3.90	558	193
C1-naphthalenes	2010	1130	<2.38	4.56	<2.38	2.74	<2.38	<2.38	<2.38	50.6	3.57	14.6	11.8	282	2930
C2-naphthalenes	7950	6380	20.7	653	6380	77.6	4.86	13.8	71	2080	16.3	530	918	1240	14600
C3-naphthalenes	11300	6480	65.9	1690	40600	99.4	17.5	49.4	165	2780	39.8	1060	1670	2900	12700
C4-naphthalenes	7580	4160	82.5	1570	60500	111	47.5	111	264	3680	115	1210	1870	4020	9510
fluorene	574	13.4	0.573	9.95	40.7	2.80	1.01	1.86	4.04	57.24	3.84	59.0	130	79.6	888
C1-fluorenes	262	30.5	0.394	27.9	306	1.88	0.533	1.23	5.33	14.8	1.89	35.1	48.5	48.3	315
C2-fluorenes	193	55	0.364	24.6	1120	2.6	1.92	3.34	6.63	15.1	4.36	39.6	62.5	86.3	271
C3-fluorenes	69.6	35.8	<1.07	11.9	672	7.23	5.98	6.63	9.85	17	6.3	20.8	40.8	62.7	163
phenanthrene	314	5.28	0.831	32.0	54.5	1.71	0.488	0.985	3.36	2.78	1.10	31.3	54.6	47.67	327
C1-phenanthrenes & anthracenes	54.1	5.76	0.233	5.29	131	1.33	0.56	0.919	1.81	1.15	0.833	10.2	11.3	15.2	66.7
C2-phenanthrenes & anthracenes	43.4	10.8	0.752	5.84	354	1.77	1.46	2.24	2.91	2.21	2.73	15.3	20	28.4	95.4
C3-phenanthrenes & anthracenes	35.4	13.7	<0.726	5.68	398	0.726	2.37	3.46	3.8	<0.726	3.73	14.5	20.9	128	219
C4-phenanthrenes & anthracenes	6.14	5.95	<1.37	1.57	229	<1.37	<1.37	<1.37	<1.37	<1.37	<1.37	6.33	10.3	<1.37	<1.37
dibenzothiophene	41.5	1.32	0.127	3.53	11.6	0.828	0.151	0.331	0.545	0.811	0.363	4.17	6.18	4.61	38.9
C1-dibenzothiophenes	2.44	1.25	0.0891	0.992	29	0.813	0.183	0.283	0.62	0.386	0.191	1.49	1.93	2.26	8.79
C2-dibenzothiophenes	3.05	0.946	<0.175	0.448	27.2	0.244	0.227	0.24	0.338	0.207	0.218	0.815	1.32	1.97	5.38
C3-dibenzothiophenes	<0.910	<0.910	<0.910	<0.910	5.3	<0.910	<0.910	<0.910	<0.910	<0.910	<0.910	<0.910	<0.910	<0.910	1.59
fluoranthene	10.8	0.165	0.0953	1.66	1.95	0.229	0.160	0.491	0.635	0.435	0.781	5.08	5.52	4.96	16.9
C1-fluoranthenes & pyrenes	2.88	0.142	0.0501	0.385	4.31	0.101	0.0803	0.22	0.205	0.163	0.304	1.86	2.02	2.11	8.8
C2-fluoranthenes & pyrenes	0.0436	<0.00424	<0.00424	<0.00424	0.13	<0.00424	<0.00424	<0.00424	<0.00424	<0.00424	<0.00424	0.00912	0.0214	0.0405	0.0442
chrysene & triphenylene	0.252	0.00828	0.00394	0.0405	0.260	0.0120	0.00717	0.0319	0.0245	0.0163	0.0462	0.305	0.290	0.305	1.48
C1-benz[a]anthracenes & chrysenes & triphenylenes	0.113	0.0227	0.0077	0.0254	0.48	0.00922	0.00624	0.0205	0.0153	0.0121	0.0274	0.133	0.119	0.158	0.643
C2-benz[a]anthracenes & chrysenes & triphenylenes	0.00863	<0.00137	<0.00137	<0.00137	0.0504	<0.00137	<0.00137	<0.00137	<0.00137	<0.00137	<0.00137	0.00808	0.0071	0.0124	0.0327
naphthalene*	54.2	<0.296	<0.296	<0.296	5.05	0.979	<0.296	1.69	0.922	<0.296	0.324	7.41	7.58	442	155
acenaphthylene*	22.1	<0.11	<0.11	<0.11	4.76	<0.11	<0.11	0.354	0.532	<0.11	<0.11	<0.11	<0.11	<0.11	<0.11
acenaphthene*	3470	26.5	9.07	306	<0.0533	42.5	10.3	11.5	67.3	1610	18.9	131	391	548	4570
fluorene*	548	13.3	0.838	10.3	47.8	2.83	1.17	1.94	3.34	456	3.55	53.8	102	65.1	564
dibenzothiophene*	27.9	0.778	0.11	2.2	7.34	0.557	0.101	0.225	0.378	0.553	0.249	2.89	4.41	3.26	28.5
phenanthrene*	296	4.26	0.664	26.8	44.2	1.52	0.47	0.878	3	2.46	0.967	27.8	49.9	43	322
anthracene*	8.36	0.408	<0.0118	<0.0118	<0.0118	0.189	0.1	0.323	0.297	0.341	0.625	5.52	<0.0118	4.41	34.6
fluoranthene*	9.14	0.128	0.0791	1.3	1.6	0.193	0.136	0.415	0.54	0.36	0.644	4.29	4.75	4.26	15.1

pyrene*	7.5	0.196	0.0934	1.03	3.59	0.191	0.153	0.382	0.491	0.327	0.612	3.33	4.4	2.69	15.2
benz[a]anthracene*	0.188	<0.000908	<0.000908	0.0275	0.136	0.000907	0.000658	0.0204	0.0166	0.0122	0.0281	0.232	0.21	0.221	1.12
chrysene*	0.163	<0.000603	<0.000603	0.03	0.187	0.00611	0.0039	0.0197	0.0158	0.00967	0.0247	0.21	0.201	0.235	1.14
benzo(b)fluoranthene*	0.0385	<0.000447	<0.000447	0.0114	0.0254	0.00468	0.00315	0.00838	0.00692	0.00528	0.0126	0.0747	0.0584	0.0932	0.336
benzo(k)fluoranthene*	0.0233	<0.000759	<0.000759	<0.000759	<0.000759	0.00495	<0.000759	0.00814	0.00667	0.0056	0.00868	0.0402	0.0298	0.051	0.177
benzo(e)pyrene*	0.034	<0.00125	<0.00125	<0.00125	0.0383	<0.00125	0.00302	0.00942	0.0076	0.00581	0.0128	0.0714	0.0544	0.0822	0.29
benzo(a)pyrene*	0.0289	<0.00172	<0.00172	<0.00172	0.0251	<0.00172	<0.00172	<0.00172	<0.00172	<0.00172	0.0084	0.0525	0.0418	1.72	0.256
perylene*	0.0362	<0.00166	<0.00166	<0.00166	0.052	0.00895	0.00423	0.00819	0.00894	<0.00166	0.00882	0.0259	0.0191	0.0307	0.108
indeno(1,2,3-c,d)pyrene*	<0.000501	<0.000501	<0.000501	<0.000501	<0.000501	<0.000501	<0.000501	<0.000501	<0.000501	<0.000501	<0.000501	<0.000501	<0.000501	0.0337	<0.000501
dibenz(a,h)anthracene*	<0.00200	<0.00200	<0.00200	<0.00200	<0.00200	<0.00200	<0.00200	<0.00200	<0.00200	<0.00200	<0.00200	<0.00200	<0.00200	<0.00200	<0.00200
benzo(ghi)perylene*	<0.000603	<0.000603	<0.000603	<0.000603	<0.000603	<0.000603	<0.000603	<0.000603	<0.000603	<0.000603	<0.000603	0.0156	0.0118	0.0282	<0.000603
triphenylene*	0.0746	<0.000548	<0.000548	0.0124	0.0762	0.00756	0.0047	0.0136	0.01	0.00786	0.0205	0.0975	0.0874	0.0796	0.313

*Compounds indicated by a * are obtained from the 63 PAH method[2]*

Table S22. Chamber Study Instrument Concentrations

	Instrument Concentration in pg/μL	
**Analyte**	**AAB3_LB**	**AAB4_RB**
naphthalene	522000	423000
C1-naphthalenes	15900	16000
C2-naphthalenes	856	844
C3-naphthalenes	<14.8	<14.8
C4-naphthalenes	<11.2	<11.2
fluorene	13100	16500
C1-fluorenes	700	884
C2-fluorenes	<9.58	<9.58
C3-fluorenes	<44.5	<44.5
phenanthrene	49800	63200
C1-phenanthrenes & anthracenes	780	1000
C2-phenanthrenes & anthracenes	<12.8	<12.8
C3-phenanthrenes & anthracenes	<66.4	<66.4
C4-phenanthrenes & anthracenes	<125	<125
dibenzothiophene	62.2	116
C1-dibenzothiophenes	<1.44	<1.44
C2-dibenzothiophenes	<1.27	<1.27
C3-dibenzothiophenes	<14.6	<14.6
fluoranthene	7250	9660
C1-fluoranthenes & pyrenes	1040	1340
C2-fluoranthenes & pyrenes	<2.04	<2.04
chrysene & triphenylene	745	676
C1-benz[a]anthracenes & chrysenes & triphenylenes	143	56.8
C2-benz[a]anthracenes & chrysenes & triphenylenes	<1.00	<1.00
naphthalene*	739000	651000
acenaphthylene*	168000	210000
acenaphthene*	1250	969
fluorene*	18500	26200
dibenzothiophene*	68.0	90.9
phenanthrene*	61000	86200
anthracene*	7680	9360
fluoranthene*	9400	13400
pyrene*	5120	7420
benz[a]anthracene*	1020	1030
chrysene*	1020	951
benzo(b)fluoranthene*	1080	820
benzo(k)fluoranthene*	385	307
benzo(e)pyrene*	840	636
benzo(a)pyrene*	1020	935
perylene*	215	179
indeno(1,2,3-c,d)pyrene*	666	479
dibenz(a,h)anthracene*	1.02	1.02
benzo(ghi)perylene*	606	459
triphenylene*	505	553

*Compounds indicated by a * are obtained from the 63 PAH method[2]*

Table S23. Deepwater Horizon Ratio Results

Abbreviation	Gulfport, MS June	Gulfport, MS June	Gulfport, MS August	Pensacola, FL August	Grand Isle, LA June	Grand Isle, LA June
A0/PA0	0.264	0.552	0.239	0.188	0.005	0.001
P0/A0	2.79	0.810	3.18	4.32	188	1220
PA1/PA0	4.28	1.25	14.7	2.57	255	84.0
PA0/PA01	0.189	0.445	0.064	0.280	0.004	0.012
FL0/PY0	0.938	1.29	0.829	1.33	0.382	0.688
FL0/FLPY	0.484	0.563	0.453	0.571	0.276	0.408
FLPY0/FLPY01	0.825	0.831	0.851	0.782	0.058	0.061
FLP1/PY0	0.410	0.464	0.320	0.649	22.6	26.0
FLP1/FLPY0	0.211	0.203	0.175	0.279	16.4	15.4
FLPY/(P2 + P3 + P4)	0.0564	0.0206	0.0558	0.0398	4.80E-05	7.56E-05
BaA/Ch0	0.470	0.574	0.318	0.197	3.06E-04	0.001
BaA/228	0.320	0.365	0.241	0.165	3.25E-04	0.001
D2/P2	0.025	0.146	0.093	0.053	0.078	0.075
D3/P3	0.026	0.044	0.039	0.023	0.020	0.018
PY0/BaP	101	324	5.55	1.16	1330	377
IP/ghi	1.06	0.052	1.01	1.16	0.047	0.046
IP/IP + ghi	0.515	0.050	0.502	0.537	0.045	0.044
BeP/BaP	2.51	2.56	14.7	11.7	6.87	76.1
∑alkyl/PAHs	26.1	440	99.7	131	10300	4900
L/H	17.1	140	1.68	0.626	2.38	8.88
LPAH/HPAH	13.6	12.9	1.15	0.623	1.70	5.70
pyrogenic index	0.005	0.001	0.016	0.018	1.04E-05	1.11E-05
Petrogenic Sum Score	8	9	8	8	**25**	**24**
Pyrogenic Sum Score	**16**	**18**	**19**	**20**	3	3

Table S24. St Helens Air Ratio Results

Abbreviation	Site 3	Site 9 Rep1	Site 9 Rep2	Site 9 Rep3	Site 12
A0/PA0	N/A	N/A	N/A	N/A	0.063
P0/A0	N/A	N/A	N/A	N/A	15.0
PA1/PA0	N/A	N/A	N/A	N/A	0.423
PA0/PA01	N/A	N/A	N/A	N/A	0.703
FL0/PY0	2.43	2.26	2.18	2.28	2.46
FL0/FLPY	0.708	0.694	0.686	0.695	0.711
FLPY0/FLPY01	0.898	0.883	0.889	0.892	0.892
FLP1/PY0	0.391	0.432	0.397	0.399	0.418
FLP1/FLPY0	0.114	0.132	0.125	0.122	0.121
FLPY/(P2 + P3 + P4)	N/A	N/A	N/A	N/A	N/A
BaA/Ch0	0.455	0.749	0.804	0.715	0.902
BaA/228	0.313	0.428	0.446	0.417	0.474
D2/P2	0.0305	0.0290	0.0310	0.0310	0.0250
D3/P3	N/A	N/A	N/A	N/A	N/A
PY0/BaP	N/A	N/A	N/A	N/A	9680
IP/ghi	N/A	N/A	N/A	N/A	N/A
IP/IP + ghi	N/A	N/A	N/A	N/A	N/A
BeP/BaP	N/A	N/A	N/A	N/A	N/A
∑alkyl/PAHs	220	120	99.2	108	340
L/H	2930	2010	4420	1650	1680
LPAH/HPAH	900	959	2110	969	187
pyrogenic index	0.00112	0.00300	0.00300	0.00300	0.00300
Petrogenic Sum Score	6	5	5	5	9
Pyrogenic Sum Score	**10**	**11**	**12**	**12**	**13**

Table S25. St Helens Water Ratio Results

Abbreviation	Site 2	Site 3	Site 4	Site 9	Site 10 Rep 1	Site 10 Rep 2	Site 10 Rep 3	Site 12	Site 13	Site 14
A0/PA0	0.096	0.278	0.248	0.330	0.206	0.221	0.205	0.235	0.317	0.093
P0/A0	9.46	2.6	3.03	3.11	3.85	3.52	3.87	3.26	2.15	2.77
PA1/PA0	0.986	2.58	2.5	1.51	1.42	1.54	1.46	1.5	1.49	1.39
PA0/PA01	0.503	0.279	0.286	0.398	0.413	0.394	0.406	0.399	0.402	0.418
FL0/PY0	0.804	0.721	0.678	0.797	0.75	0.762	0.73	0.722	0.756	0.718
FL0/FLPY	0.446	0.419	0.404	0.444	0.429	0.432	0.422	0.419	0.431	0.418
FLPY0/FLPY01	0.848	0.634	0.8	0.804	0.807	0.811	0.806	0.78	0.774	0.838
FLP1/PY0	0.323	0.993	0.42	0.439	0.418	0.412	0.416	0.485	0.512	0.331
FLP1/FLPY0	0.179	0.577	0.25	0.244	0.239	0.234	0.24	0.282	0.292	0.193
FLPY/(P2 + P3 + P4)	N/A	0.0978	0.102	0.117	0.125	0.129	N/A	0.122	0.135	0.118
BaA/Ch0	N/A	0.503	0.526	0.579	0.612	0.602	0.558	0.625	0.652	0.481
BaA/228	0.384	0.334	0.345	0.367	0.38	0.376	0.358	0.385	0.395	0.325
D2/P2	0.05	0.054	0.0503	0.0588	0.0554	0.0585	0.0553	0.0522	0.0569	0.0399
D3/P3	0.0147	0.0145	0.0125	0.0195	0.012	0.0188	0.0135	0.0112	0.0109	0.00946
PY0/BaP	279	154	166	158	179	177	186	139	110	188
IP/ghi	N/A	N/A	N/A	0.784	0.769	0.693	0.79	N/A	N/A	N/A
IP/IP + ghi	N/A	N/A	N/A	0.439	0.435	0.409	0.442	N/A	N/A	N/A
BeP/BaP	2.52	2.69	2.69	2.16	2.02	1.8	1.88	1.74	1.66	1.93
∑alkyl/PAHs	65.9	240	227	149	126	131	142	135	92.6	132
L/H	30.7	19.4	17.8	24.9	26.5	26.7	28	21.9	17.6	32.6
LPAH/HPAH	3.56	1.4	1.3	2.16	3.14	2.69	2.92	1.9	1.62	3.32
pyrogenic index	0.0108	0.00377	0.00381	0.00765	0.00865	0.00805	0.0076	0.00752	0.0106	0.00845
Petrogenic Sum Score	9	8	8	8	6	10	6	8	5	9
Pyrogenic Sum Score	**13**	**15**	**16**	**17**	**17**	**17**	**16**	**17**	**17**	**13**

Table S26. St Helens Shallow Porewater Ratio Results

Abbreviation	Site 1	Site 2	Site 3	Site 4	Site 5	Site 6	Site 7	Site 8	Site 9	Site 10	Site 11	Site 12	Site 13	Site 14	Site 15
A0/PA0	0.289	0.118	N/A	0.148	0.251	0.291	0.271	0.330	0.330	0.359	0.429	0.0425	0.562	0.251	0.279
P0/A0	2.46	7.48	N/A	5.76	2.98	2.44	2.69	2.03	2.03	1.79	1.33	22.5	0.780	2.98	2.58
PA1/PA0	0.794	2.23	N/A	1.19	0.706	0.662	0.746	0.662	0.661	0.793	0.630	0.181	0.730	0.593	0.660
PA0/PA01	0.557	0.309	N/A	0.456	0.586	0.602	0.573	0.602	0.602	0.558	0.613	0.847	0.578	0.628	0.602
FL0/PY0	1.05	0.286	0.811	0.738	0.901	1.06	0.945	0.945	1.15	0.990	1.02	1.43	1.02	1.06	1.18
FL0/FLPY	0.512	0.222	0.448	0.425	0.474	0.515	0.486	0.486	0.534	0.497	0.505	0.589	0.504	0.516	0.541
FLPY0/FLPY01	0.815	0.548	0.781	0.825	0.788	0.766	0.800	0.797	0.790	0.834	0.799	0.881	0.811	0.827	0.805
FLP1/PY0	0.465	1.06	0.508	0.369	0.512	0.629	0.488	0.496	0.569	0.397	0.509	0.327	0.472	0.432	0.528
FLP1/FLPY0	0.227	0.824	0.281	0.212	0.269	0.305	0.251	0.255	0.265	0.199	0.252	0.134	0.234	0.209	0.242
FLPY/(P2 + P3 + P4)	0.135	0.00434	N/A	N/A	0.0581	N/A	N/A	0.113	N/A	N/A	N/A	0.652	0.221	N/A	N/A
BaA/Ch0	0.645	0.503	N/A	0.585	0.328	0.557	0.570	0.554	0.569	0.610	0.642	0.393	0.059	0.637	0.612
BaA/228	0.392	0.335	N/A	0.369	0.247	0.358	0.363	0.356	0.363	0.379	0.391	0.282	0.056	0.389	0.380
D2/P2	0.100	0.0731	0.177	0.116	0.076	0.119	0.120	0.070	0.107	0.079	0.083	0.0618	0.066	0.126	0.085
D3/P3	N/A	0.0195	N/A	N/A	N/A	N/A	N/A	N/A	N/A	N/A	N/A	N/A	N/A	N/A	N/A
PY0/BaP	N/A	N/A	N/A	N/A	N/A	N/A	N/A	127	N/A	138	81.6	159	99.7	66.5	43.2
IP/ghi	N/A	N/A	N/A	N/A	N/A	N/A	N/A	N/A	N/A	N/A	N/A	N/A	N/A	N/A	N/A
IP/IP + ghi	N/A	N/A	N/A	N/A	N/A	N/A	N/A	N/A	N/A	N/A	N/A	N/A	N/A	N/A	N/A
BeP/BaP	N/A	N/A	N/A	N/A	N/A	N/A	N/A	1.94	N/A	1.44	1.67	1.90	1.56	1.39	1.39
∑alkyl/PAHs	34.9	844	N/A	20.4	22.6	16.2	14.2	22.0	15.0	41.5	21.8	3.63	30.5	12.5	23.4
L/H	47.6	345	N/A	118	45.3	26.9	41.9	42.0	27.6	42.5	27.8	113	4.14	41.9	18.0
LPAH/HPAH	21.2	168	N/A	37.8	68.5	25.5	30.0	21.1	23.3	21.4	15.2	35.9	6.38	28.7	13.9
pyrogenic index	0.037	0.0008	N/A	0.028	0.009	0.036	0.035	0.033	0.058	0.034	0.049	0.0363	0.047	0.054	0.040
Petrogenic Sum Score	5	11	1	5	5	5	5	6	3	6	6	9	8	4	6
Pyrogenic Sum Score	**20**	12	**8**	**12**	**16**	**19**	**17**	**18**	**19**	**18**	**19**	12	**18**	**19**	**19**

Table S27. St Helens Deep Porewater Ratio Results

Abbreviation	Site 1	Site 2	Site 3	Site 4	Site 5	Site 6	Site 7	Site 8	Site 9	Site 10	Site 11	Site 12	Site 13	Site 14	Site 15
A0/PA0	0.0274	0.087	N/A	N/A	N/A	0.111	0.176	0.269	0.0902	0.122	0.393	0.166	N/A	0.093	0.0973
P0/A0	35.5	10.4	N/A	N/A	N/A	8.045	4.692	2.72	10.1	7.22	1.55	5.03	N/A	9.75	9.28
PA1/PA0	0.178	1.23	N/A	N/A	N/A	0.776	0.982	0.766	0.549	0.411	0.519	0.303	N/A	0.320	0.187
PA0/PA01	0.849	0.448	N/A	N/A	N/A	0.563	0.505	0.566	0.646	0.709	0.658	0.767	N/A	0.758	0.842
FL0/PY0	1.22	0.654	0.848	1.27	0.446	1.011	0.891	1.09	1.10	1.10	1.05	1.29	1.08	1.59	0.994
FL0/FLPY	0.550	0.395	0.459	0.559	0.308	0.503	0.471	0.521	0.524	0.524	0.513	0.563	0.519	0.613	0.499
FLPY0/FLPY01	0.852	0.695	0.775	0.858	0.546	0.792	0.782	0.784	0.834	0.808	0.807	0.806	0.819	0.767	0.775
FLP1/PY0	0.384	0.725	0.536	0.374	1.20	0.530	0.526	0.576	0.418	0.499	0.492	0.552	0.458	0.786	0.580
FLP1/FLPY0	0.173	0.438	0.290	0.165	0.831	0.263	0.278	0.276	0.199	0.238	0.240	0.241	0.220	0.304	0.291
FLPY/(P2 + P3 + P4)	0.196	0.0107	N/A	0.178	0.00529	N/A	N/A	N/A	N/A	N/A	N/A	0.213	0.179	N/A	N/A
BaA/Ch0	0.793	N/A	N/A	0.649	0.517	N/A	N/A	0.610	0.641	0.699	0.622	0.754	0.727	0.704	0.767
BaA/228	0.442	N/A	N/A	0.394	0.341	N/A	N/A	0.379	0.391	0.411	0.383	0.430	0.421	0.413	0.434
D2/P2	0.0703	0.087	N/A	0.0767	0.0769	0.138	0.155	0.107	0.116	0.094	0.080	0.053	0.066	0.069	0.0564
D3/P3	N/A	N/A	N/A	N/A	0.0133	N/A	N/A	N/A	N/A	N/A	N/A	N/A	N/A	N/A	0.0073
PY0/BaP	259	N/A	N/A	N/A	143	N/A	N/A	N/A	N/A	N/A	72.8	63.5	105	1.56	59.3
IP/ghi	N/A	N/A	N/A	N/A	N/A	N/A	N/A	N/A	N/A	N/A	N/A	N/A	N/A	1.19	N/A
IP/IP + ghi	N/A	N/A	N/A	N/A	N/A	N/A	N/A	N/A	N/A	N/A	N/A	N/A	N/A	0.544	N/A
BeP/BaP	1.18	N/A	N/A	N/A	1.53	N/A	N/A	N/A	N/A	N/A	1.52	1.36	1.30	0.048	1.13
∑alkyl/PAHs	76.3	N/A	42.6	134	1840	40.9	15.7	23.2	54.2	2340	24.6	53.5	63.5	17.2	73.1
L/H	671	N/A	N/A	390	116	99.0	81.1	30.9	84.0	92.6	31.0	63.1	101	22.9	129
LPAH/HPAH	257	N/A	N/A	142	18.8	127	52.8	23.9	72.9	2840	21.0	27.5	56.7	118	168
pyrogenic index	0.117	N/A	N/A	0.0766	0.000093	0.136	0.115	0.064	0.127	0.177	0.102	0.048	0.084	0.063	0.112
Petrogenic Sum Score	10	5	1	3	9	3	3	3	6	3	4	6	4	6	7
Pyrogenic Sum Score	**15**	**8**	**6**	**14**	9	**14**	**15**	**19**	**13**	**16**	**19**	**17**	**12**	**17**	**12**

Table S28. Chamber Study Ratio Results

Abbreviation	AAB3_LB	AAB4_RB
A0/PA0	0.112	0.098
P0/A0	7.94	9.21
PA1/PA0	0.011	0.010
PA0/PA01	0.989	0.990
FL0/PY0	1.84	1.81
FL0/FLPY	0.647	0.644
FLPY0/FLPY01	0.933	0.940
FLP1/PY0	0.203	0.181
FLP1/FLPY0	0.072	0.064
FLPY/(P2 + P3 + P4)	N/A	N/A
BaA/Ch0	1.00	1.08
BaA/228	0.401	0.406
D2/P2	N/A	N/A
D3/P3	N/A	N/A
PY0/BaP	5.02	7.94
IP/ghi	1.10	1.04
IP/IP + ghi	0.524	0.511
BeP/BaP	0.824	0.680
∑alkyl/PAHs	0.023	0.026
L/H	17.6	23.1
LPAH/HPAH	47.8	37.3
pyrogenic index	11.8	14.5
Petrogenic Sum Score	2	5
Pyrogenic Sum Score	**21**	**19**

Table S29. PAH Ratio Accuracy in Source Prediction for SRMs and Known PAH Source Samples

	Standard Reference Materials					Environmental Samples			
**Ratio**	**SRM 2779**	**SRM 1580**	**SRM 1582**	**SRM 1597a**	**SRM 1975**	**DWH**	**St. Helens Water**	**St Helens Porewater**	**Chamber**
**A0/PA0**	N/A	✓	N/A	✓	N/A	✓	✓	✓	✓
**P0/A0**	N/A	✓	N/A	✓	N/A	✓	✓	✓	✓
**PA1/PA0**	N/A	✓	N/A	✓	N/A	✓	X	✓	✓
PA0/PA01	N/A	✓	N/A	✓	N/A	✓	X	✓	✓
FL0/PY0	✓	*	*	✓	✓	✓	*	✓	✓
FL0/FLPY	✓	✓	*	✓	✓	✓	*	✓	✓
**FLPY0/FLPY01**	✓	✓	✓	✓	✓	✓	✓	✓	✓
FLP1/PY0	✓	✓	*	✓	*	✓	*	*	✓
FLP1/FLPY0	✓	✓	✓	✓	✓	✓	✓	✓	✓
FLPY/(P2 + P3 + P4)	N/A	✓	X	N/A	N/A	X	✓	✓	✓
BaA/Ch0	✓	✓	*	✓	X	✓	✓	✓	✓
BaA/228	✓	✓	✓	✓	X	✓	✓	✓	✓
D2/P2	X	✓	X	N/A	✓	X	✓	✓	N/A
D3/P3	X	✓	X	✓	✓	X	✓	*	N/A
PY0/BaP	X	✓	N/A	X	N/A	✓	X	X	✓
IP/ghi	N/A	*	✓	*	N/A	✓	*	*	✓
IP/IP + ghi	N/A	*	✓	*	N/A	✓	*	*	✓
BeP/BaP	X	*	N/A	*	N/A	✓	*	*	✓
∑alkyl/PAHs	✓	✓	✓	✓	✓	✓	X	X	✓
L/H	✓	✓	✓	X	X	✓	X	X	X
LPAH/HPAH	✓	✓	✓	*	*	✓	X	X	X
**pyrogenic index**	✓	*	*	*	*	✓	X	X	X

✓: *ratio correctly predicted the PAH source*
"X": ratio incorrectly predicted the PAH source
"N/A": at least one compound in the ratio below limit of detection, ratio not calculated
*: *ratio value falls in the mixed range and cannot be assigned to a particular signature*

References

1. U.S. Environmental Protection Agency. Estimation Programs Interface Suite™ for Microsoft® Windows, v 4.1.25. Washington, DC, USA: United States Environmental Protection Agency; 2015.
2. Anderson KA, Szelewski MJ, Wilson G, Quimby BD, Hoffman PD. Modified ion source triple quadrupole mass spectrometer gas chromatograph for polycyclic aromatic hydrocarbon analyses. Journal of Chromatography A. 2015;1419:89-98. doi:https://doi.org/10.1016/j.chroma.2015.09.054.
